# The annotation of *GBA1* has been concealed by its protein-coding pseudogene *GBAP1*

**DOI:** 10.1126/sciadv.adk1296

**Published:** 2024-06-26

**Authors:** Emil K. Gustavsson, Siddharth Sethi, Yujing Gao, Jonathan W. Brenton, Sonia García-Ruiz, David Zhang, Raquel Garza, Regina H. Reynolds, James R. Evans, Zhongbo Chen, Melissa Grant-Peters, Hannah Macpherson, Kylie Montgomery, Rhys Dore, Anna I. Wernick, Charles Arber, Selina Wray, Sonia Gandhi, Julian Esselborn, Cornelis Blauwendraat, Christopher H. Douse, Anita Adami, Diahann A. M. Atacho, Antonina Kouli, Annelies Quaegebeur, Roger A. Barker, Elisabet Englund, Frances Platt, Johan Jakobsson, Nicholas W. Wood, Henry Houlden, Harpreet Saini, Carla F. Bento, John Hardy, Mina Ryten

**Affiliations:** ^1^ Genetics and Genomic Medicine, Great Ormond Street Institute of Child Health, University College London, London, UK.; ^2^ Aligning Science Across Parkinson’s (ASAP) Collaborative Research Network, Chevy Chase, MD 20815, USA.; ^3^ Astex Pharmaceuticals, 436 Cambridge Science Park, Cambridge, UK.; ^4^ NIHR Great Ormond Street Hospital Biomedical Research Centre, University College London, London, UK.; ^5^ Laboratory of Molecular Neurogenetics, Department of Experimental Medical Science, Wallenberg Neuroscience Center and Lund Stem Cell Center, Lund, Sweden.; ^6^ Department of Clinical and Movement Neurosciences, UCL Queen Square Institute of Neurology, University College London, London, UK.; ^7^ The Francis Crick Institute, London, UK.; ^8^ Department of Neurodegenerative Disease, UCL Queen Square Institute of Neurology, University College London, London, UK.; ^9^ Laboratory of Neurogenetics, National Institute on Aging, National Institutes of Health, Bethesda, MD 20892, USA.; ^10^ Laboratory of Epigenetics and Chromatin Dynamics, Department of Experimental Medical Science, Lund Stem Cell Center, Lund University, Lund, Sweden.; ^11^ Wellcome-MRC Cambridge Stem Cell Institute and John Van Geest Centre for Brain Repair, Department of Clinical Neurosciences, University of Cambridge, Cambridge, UK.; ^12^ Department of Clinical Neurosciences, University of Cambridge, Clifford Albutt Building, Cambridge, UK.; ^13^ Department of Neuropathology, University of Lund, Lund, Sweden.; ^14^ Department of Pharmacology, University of Oxford, Oxford, UK.; ^15^ Department of Neuromuscular Disease, UCL Queen Square Institute of Neurology, UCL, London, UK.; ^16^ Reta Lila Weston Institute, UCL Queen Square Institute of Neurology, UCL, London, UK.; ^17^ UK Dementia Research Institute at UCL, UCL Queen Square Institute of Neurology, UCL, London, UK.; ^18^ NIHR University College London Hospitals Biomedical Research Centre, London, UK.; ^19^ Institute for Advanced Study, The Hong Kong University of Science and Technology, Hong Kong SAR, China.

## Abstract

Mutations in *GBA1* cause Gaucher disease and are the most important genetic risk factor for Parkinson’s disease. However, analysis of transcription at this locus is complicated by its highly homologous pseudogene, *GBAP1*. We show that >50% of short RNA-sequencing reads mapping to *GBA1* also map to *GBAP1*. Thus, we used long-read RNA sequencing in the human brain, which allowed us to accurately quantify expression from both *GBA1* and *GBAP1*. We discovered significant differences in expression compared to short-read data and identify currently unannotated transcripts of both *GBA1* and *GBAP1*. These included protein-coding transcripts from both genes that were translated in human brain, but without the known lysosomal function—yet accounting for almost a third of transcription. Analyzing brain-specific cell types using long-read and single-nucleus RNA sequencing revealed region-specific variations in transcript expression. Overall, these findings suggest nonlysosomal roles for *GBA1* and *GBAP1* with implications for our understanding of the role of *GBA1* in health and disease.

## Teaser

Long-read RNA sequencing uncovers unexpected protein-coding roles for *GBA1* and *GBAP1*, exhibiting tissue and cell type selectivity.

## INTRODUCTION

The human genome contains regions that evade comprehensive analysis through short-read sequencing technologies and thus remain poorly studied. While these difficulties can be attributed to challenges with sequencing (e.g., high GC content), they are most commonly the result of duplicated genomic regions (*1*). This leads to sequencing reads aligning to multiple genomic locations due to a high degree of sequence similarity, a phenomenon known as multimapping. Given that defective gene copies with high sequence similarity to their parent genes, termed pseudogenes, are frequently found in the human genome, this is a common problem (*2*).

While the impact of multimapping has been investigated in the context of pathogenic variant detection and can cause variants to be “missed” using conventional analyses (*3*), the effect of multimapping on transcriptomic analyses has received less attention despite the problem being similar in nature (*4*). This is surprising given the considerable number of genes affected, many of which are implicated in human disease. Short-read RNA sequencing (RNA-seq) has been crucial to our understanding of transcript annotation, gene expression, and its tissue and cell type–specific regulation. However, a major challenge in analyzing these datasets is the difficulty of annotating parent-pseudogene pairs due to reads that cannot unambiguously map to either the parent gene or pseudogene, and so accurately quantifying gene expression.

Here, we focused on the disease-relevant example of *GBA1* and its expressed pseudogene *GBAP1*. *GBA1* encodes glucocerebrosidase (GCase), a lysosomal hydrolase (*5*) that degrades the glycosphingolipid, glucosylceramide (*6*). Biallelic mutations in *GBA1* result in decreased GCase activity causing Gaucher disease (GD) with glycosphingolipid excess in the brain and soma (*7*–*11*). Notably, family members of adults with GD face an increased risk of developing Parkinson’s disease (PD) (*12*). Furthermore, heterozygous mutations in *GBA1* are among the most important genetic risk factors for PD (*13*–*16*), contributing to a more rapid progression of motor and nonmotor symptoms (*17*–*21*), and they also appear to be important predictors for nonmotor symptom progression after deep brain stimulation surgery in patients with PD (*18*, *22*). Adding to this intricate landscape, it is noteworthy that “mild” and “severe” heterozygous *GBA1* mutations exhibit differential impacts on the risk and age at onset (AAO) of PD (*23*).

**Fig. 1.**
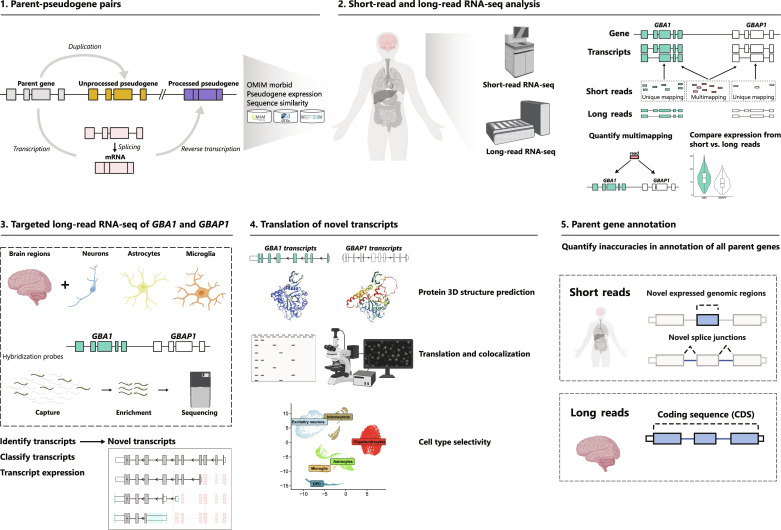
Scheme of data generation and analysis overview. Schematics outlining the methodological framework used in this study.

To address the limitations of short sequencing reads, which seldom span multiple splice junctions (*24*), we used long-read RNA-seq to examine human brain regions and induced pluripotent stem cell (iPSC)-derived brain cells in depth. Our focus was on *GBA1* and *GBAP1*, and we discovered significant differences in gene expression compared to short-read RNA-seq. Moreover, we identified a large number of novel transcripts from both genes, comprising novel protein-coding transcripts. We supported these findings by integrating short-read RNA-seq data, biochemistry, and proteomic data, which validated the novel protein-coding transcripts and confirmed that *GBAP1* is translated in cells and human brain. Furthermore, we used both long-read sequencing and annotation-agnostic short-read sequencing data and found that inaccuracies in annotation are common among parent genes. Figure 1 summarizes our analyses.

## RESULTS

### Pseudogenes are commonly expressed and alternatively spliced across human tissues

**Fig. 2.**
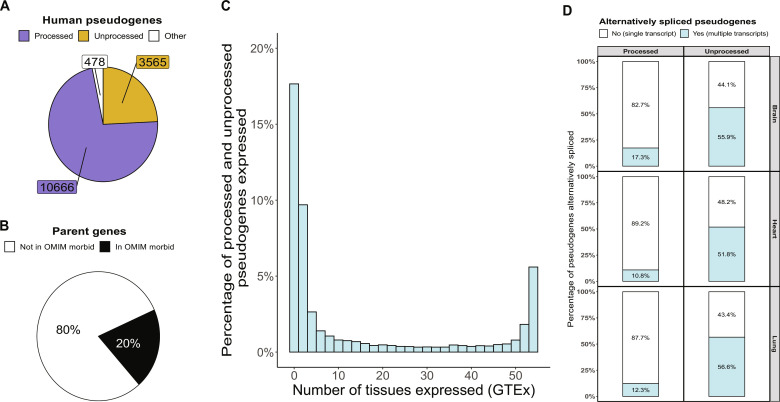
Pseudogenes are frequent and expressed across human tissues. (**A**) Pie chart showing the number of annotated pseudogenes that represent processed, unprocessed, or other pseudogenes. Other pseudogenes include unitary, IG (inactivated immunoglobulin), and TR (T cell receptor) pseudogenes. (**B**) Pie chart depicting the percentage of parent genes that are OMIM disease genes (https://omim.org). (**C**) Histogram showing tissue expression of pseudogenes as assessed using uniquely mapping reads (generated by the GTEx Consortium, v8). (**D**) Stacked bar chart depicting alternative splicing of pseudogenes using untargeted long-read RNA-seq data from ENCODE (https://www.encodeproject.org/rna-seq/long-read-rna-seq/), including 29 samples from brain (*n* = 9), heart (*n* = 16), and lung (*n* = 6).

We started by quantifying pseudogenes from GENCODE (v38) annotation to investigate their impact on transcriptomic analyses. We identified a total of 14,709 pseudogenes in the human genome (*2*, *25*), which can be divided into processed pseudogenes (*n* = 10,666) and unprocessed pseudogenes (*n* = 3565), derived from retrotransposition of processed mRNAs and segmental duplications, respectively (Fig. 2A). To date, 10,370 pseudogenes have been confidently assigned to 3665 unique parent genes (table S1) (*26*). We found that 734 (20.0%; Fig. 2B) parent genes were linked to 1015 Online Mendelian Inheritance in Man (OMIM) phenotypes, accounting for 17.0% of all OMIM disease genes (https://omim.org/) (*27*).

To examine pseudogene expression across tissues, we used uniquely mapped short-read RNA-seq data generated by the Genotype-Tissue Expression (GTEx) (*28*, *29*) Consortium (v8, accessed 10 November 2021). We found that 64.7% of pseudogenes are expressed in ≥1 tissue (Fig. 2C) and that, on average, 25.7 ± 2.5% of pseudogenes are expressed per tissue (*n* = 41; fig. S1). We then assessed the percentage of expressed pseudogenes that are alternatively spliced (>1 transcript expressed) across human brain, heart, and lung samples using publicly available long-read RNA-seq data. On average, we found that 54.8 ± 2.6% of unprocessed pseudogenes and 13.5 ± 3.4% of processed pseudogenes are alternatively spliced (Fig. 2D). Together, this is consistent with the observation that a proportion of pseudogenes are of functional importance (*30*).

### Multimapping results in significant underestimation of *GBA1* expression in human brain

**Fig. 3.**
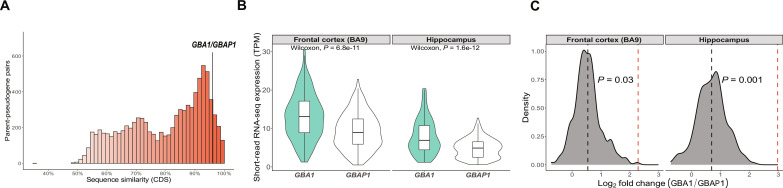
High sequence similarity causes inaccuracies in *GBA1* and *GBAP1* expression. (**A**) Histogram depicting sequence similarity of parent-pseudogene pairs across coding sequences (CDSs). *GBA1* and *GBAP1* 96% sequence similarity. (**B**) Expression in transcripts per million (TPM) of *GBA1* and *GBAP1* from GTEx using gene-level expression measures (10 November 2021, v8). (**C**) Density plot of log_2_ fold change of *GBA1* (numerator) and *GBAP1* (denominator) from GTEx using gene-level expression measures (10 November 2021, v8). The black dotted line represents the mean log_2_ fold change of *GBA1* and *GBAP1* using GTEx-derived data, while the red dotted line represents the log_2_ fold change generated through direct cDNA Oxford Nanopore Technologies (ONT) sequencing from pooled human frontal cortex (*n* = 26) and hippocampus (*n* = 27) (total library size: 42.7 million and 48.04 million reads, respectively).

We next examined the sequence similarity between pseudogenes and their parent genes as a way to investigate the potential functionality and complicating effects of the widespread expression and alternative splicing of pseudogenes. Our findings revealed that pseudogenes share an average of 80.0 ± 13.4% sequence similarity to the coding sequence (CDS) with their parent genes (Fig. 3A). As a result, genomic regions containing pseudogenes have the potential to confound transcriptomic analyses in all human tissues for a considerable proportion of protein-coding genes, including many that are causally linked to disease.

To explore this hypothesis in detail, we focused on the parent-pseudogene pair, *GBA1*-*GBAP1* (*31*). This choice was driven by the following: (i) the high sequence similarity of *GBA1-GBAP1* of 96%, which we reasoned would make both genes prone to inaccuracies in gene expression measures and transcript annotation (Fig. 3A); (ii) *GBAP1*’s broad tissue expression (determined using RNA-seq data provided by GTEx), which means that simply masking its specific genomic region during mapping would be incorrect (fig. S2); and (iii) *GBA1* has been extensively studied due to its widely known role in disease, and its pseudogene is well recognized.

We began by studying *GBA1* and *GBAP1* expression using gene-level measures from human tissues (*n* = 41) available through GTEx. Counter to previous reverse transcription polymerase chain reaction (RT-PCR)–based quantifications showing that *GBA1* is expressed at significantly higher levels than *GBAP1* (*32*), we found *GBA1* and *GBAP1* expression to be equivalent in many tissues (fig. S3), including the human brain (log_2_ fold change = 0.9 ± 0.5) (Fig. 3B). We questioned whether this observation could be explained by multimapping reads, which are often discarded in standard processing and so do not contribute to gene-level quantification of expression in many publicly available datasets [e.g., GTEx (*28*), PsychENCODE (*33*), and recount3 (*34*)]. To explore this question, we reanalyzed publicly available short-read RNA-seq of human anterior cingulate cortex samples derived from 18 individuals (*n*, control = 5, PD, with or without dementia = 13) (*35*). Using this high-depth dataset [100–base pair (bp) paired-end reads, with a mean depth of 182.9 ± 14.9 million read pairs per sample], we assessed the proportion of reads that uniquely mapped to *GBA1*. We found that only 41.7 ± 11.2% of all reads mapped to *GBA1* were uniquely mapped (fig. S4A), with 96.0 ± 2.0% of multimapped reads also aligning to *GBAP1* (fig. S4B). Considering that most reads mapped to *GBA1* and *GBAP1* are not used for quantification, we concluded that long-read RNA-seq would be required to assess their relative expression. Therefore, we applied direct cDNA Oxford Nanopore sequencing [Oxford Nanopore Technologies (ONT)] to pooled human frontal lobe (*n* individuals = 26) and hippocampus samples (*n* individuals = 27) (total library size: 42.7 million and 48.0 million reads, respectively) and found higher expression of *GBA1* (numerator) compared to *GBAP1* (denominator) (frontal lobe, log_2_ fold change = 2.3; hippocampus, log_2_ fold change = 3.1). That is, quantification with short-read RNA-seq wrongly estimated the relative expression of this parent-pseudogene pair by a 2- to 3-log_2_ fold difference (frontal cortex, Grubbs’ test statistic = 3.58, *P* = 0.03; hippocampus, Grubbs’ test statistic = 4.27, *P* < 0.01, Grubbs test for one outlier) (Fig. 3C).

### Long-read RNA-seq reveals unannotated transcripts for *GBA1* and *GBAP1* with no dominant transcript in the human brain

**Fig. 4.**
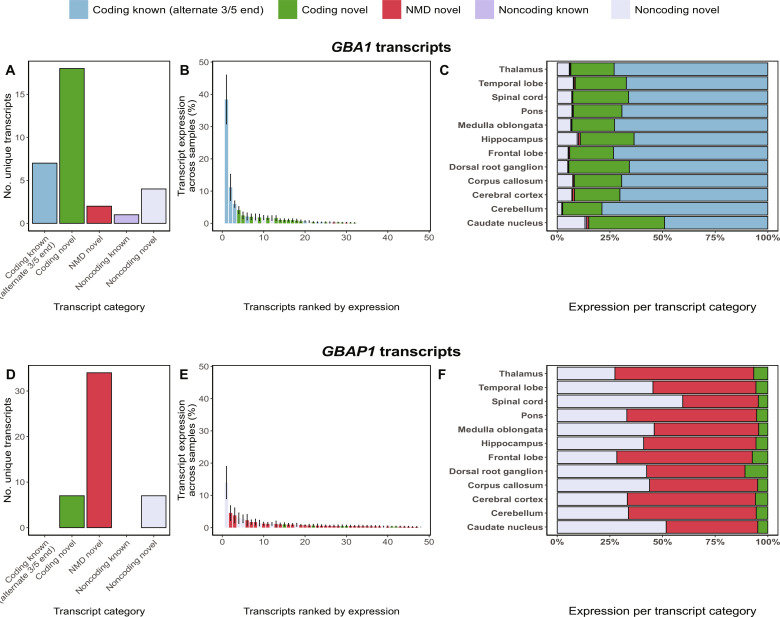
Targeted long-read RNA-seq of *GBA1* and *GBAP1* identifies frequent novel transcription. (**A**) Bar chart depicting the number of unique *GBA1* transcripts identified per transcript category through targeted long-read RNA-seq across 12 human brain regions. (**B**) Normalized expression per *GBA1* transcript corresponding to the percentage of expression per transcript out of total expression of the loci. (**C**) Stacked bar chart showing expression per transcript category of *GBA1* across 12 human brain regions. (**D**) Bar chart depicting the number of unique *GBAP1* transcripts identified per transcript category through targeted long-read RNA-seq across 12 human brain regions. (**E**) Normalized expression per *GBAP1* transcript corresponding to the percentage of expression per transcript out of total expression of the loci. (**F**) Stacked bar chart showing the expression per transcript category of *GBAP1* across 12 human brain regions.

The inaccuracies in quantification suggested that high dependence on short-read RNA-seq technologies may have also led to inaccuracies in *GBA1* and *GBAP1* transcript structures. To address this, we performed targeted Pacific Biosciences (PacBio) isoform sequencing (Iso-Seq) (fig. S5A) on 12 human brain regions. Brain tissue was used because of *GBA1*’s importance in neurological disease (*13*–*16*, *36*, *37*) and previous evidence suggesting that transcriptome annotation is most incomplete in human brain (*38*). We used PacBio Iso-Seq, which has >99% base pair accuracy enabled by circular consensus sequencing (CCS), which in turn, allows accurate mapping. To ensure that full-length reads were generated from mature mRNA alone, we used high-quality polyadenylated RNA (RNA integrity number > 8) pooled from multiple individuals per tissue (table S2). *GBA1* and *GBAP1* cDNAs were enriched using biotinylated hybridization probes designed against exonic and intronic genic regions (fig. S6) to ensure that few assumptions were made regarding transcript structure. Collapsing mapped reads resulted in 2368 *GBA1* and 3083 *GBAP1* unique transcripts, each supported by ≥2 full-length HiFi reads across all samples (fig. S7, A and B). After QC (quality control) and filtering for a minimum of 0.3% transcript usage per sample (equating to a mean of 43.4 to 11,127.2 and 15.4 to 1161.3 full-length HiFi reads for *GBA1* and *GBAP1*, respectively), we identified 32 *GBA1* and 48 *GBAP1* transcripts (Fig. 4), thus providing the most reliable annotation of *GBA1* and *GBAP1* transcription to date.

Next, we examined the identified transcripts for coding potential, nonsense-mediated decay (NMD) and similarity with the existing annotation from GENCODE to categorize transcripts into the following five categories: (i) coding known (alternate 3′/5′ end), (ii) coding novel, (iii) NMD novel, (iv) noncoding known, and (v) noncoding novel (Materials and Methods and fig. S5B). We noted that 24 of the 32 identified *GBA1* transcripts and all 48 identified *GBAP1* transcripts were absent from GENCODE (Fig. 4, A and D).

Contrary to the expectation that most protein-coding genes express one dominant transcript (*39*–*41*), we did not find a dominant *GBA1* or *GBAP1* transcript across any of the 12 brain regions sequenced. The most highly expressed *GBA1* transcript (PB.845.2786; a full splice match to ENST00000368373) only corresponded to a mean of 38.4 ± 7.6% of total transcription at the locus (Fig. 4B). Although less surprising for a pseudogene, the most highly expressed transcript of *GBAP1* (noncoding novel) only corresponded to a mean of 14.0 ± 5.0% of total transcription at the locus (Fig. 4E).

### Collectively 25 novel protein-coding transcripts of *GBA1* and its pseudogene *GBAP1* are identified

We found that of all the coding transcripts detected, 18 *GBA1* transcripts had a novel open reading frame (ORF) and 7 *GBAP1* transcripts were predicted to encode a protein, despite *GBAP1* being classified as a pseudogene (Fig. 4, A and D). Since usage of unannotated 5′ transcription start sites (TSSs) was a common feature of *GBA1* and *GBAP1* transcripts with novel ORFs (fig. S8), we focused on validating these sites using cap analysis gene expression (CAGE) peaks [defined by FANTOM5 (*42*, *43*)]. We found that, despite the fact that CAGE sequencing only captures the first 20 to 30 nucleotides from the 5′-end (unique mapping only), 57% (*n* = 4) and 50% (*n* = 9) of novel *GBA1* and *GBAP1* 5′ TSSs, respectively, were located within 50 bp of CAGE peaks, providing additional confidence in calling of these transcripts. Moreover, we validated all novel ORFs through additional targeted Iso-Seq of *GBA1* and *GBAP1* in iPSC-derived cortical neurons (*n* = 6), astrocytes (*n* = 3), and microglia (*n* = 3). In summary, we were able to detect *GBA1* and *GBAP1* transcripts with novel ORFs using a different RNA-seq technology and validate them in an independent dataset.

**Fig. 5.**
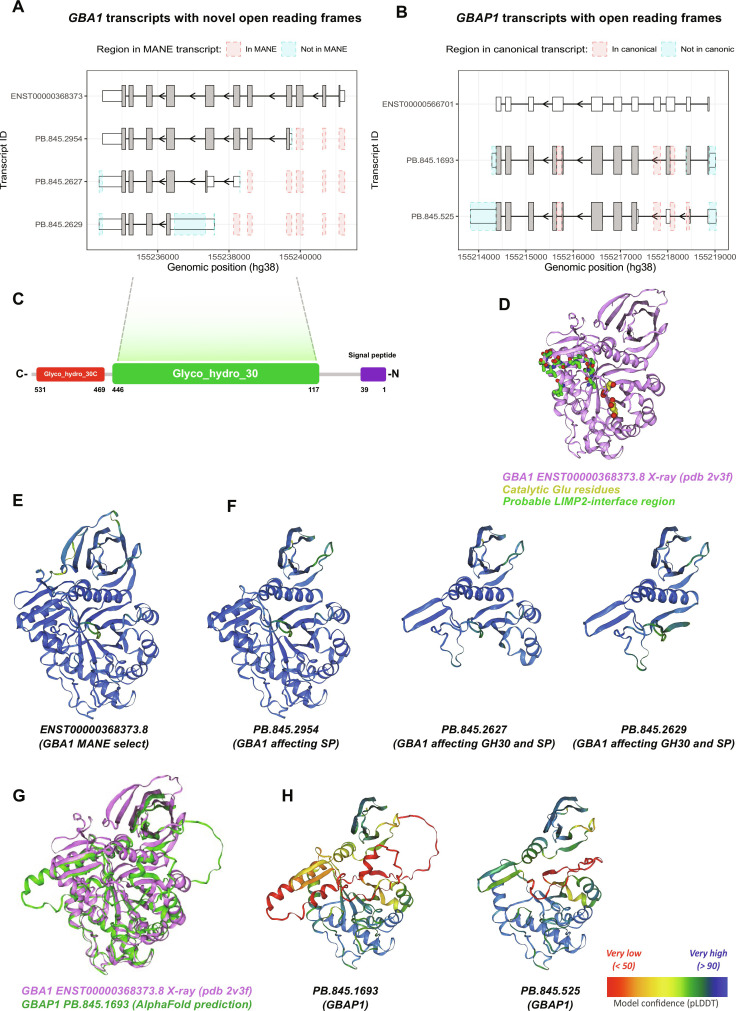
Novel proteincoding transcripts of *GBA1* and *GBAP1* share a similar structure at the C terminus but with partial or full loss of key domains. (**A**) Novel coding *GBA1* transcripts plotted using ggtranscript with differences as compared to MANE select (ENST00000368373) highlighted in blue and red. (**B**) Novel predicted coding *GBAP1* transcripts plotted using ggtranscript with differences as compared to ensemble canonical (ENST00000566701) highlighted in blue and red. (**C**) Schematic representation of GBA1 with the signal peptide (amino acids 1 to 39), glyco_hydro_30 (amino acids 117 to 446), and glycol_hydro_30C (amino acids 469 to 531). (**D**) X-ray structure of GBA1 (PDB 2v3f), with catalytic Glu residues highlighted in yellow and probable LIMP-2 interface region highlighted in purple. (**E**) AlphaFold2 predictions of *GBA1* MANE select (ENST00000368373) and (**F**) the three most highly expressed novel protein-coding GBA1 isoforms colored by prediction confidence score (pLDDT). (**G**) X-ray structure of GBA1 (PDB 2v3f) (violet) superimposed on AlphaFold2 predicted structure of the longer ORF generated by *GBAP1* PB.845.1693 (green). (**H**) AlphaFold2 predictions of the two most highly expressed novel protein-coding GBAP1 isoforms colored by prediction confidence score (pLDDT).

**Fig. 6.**
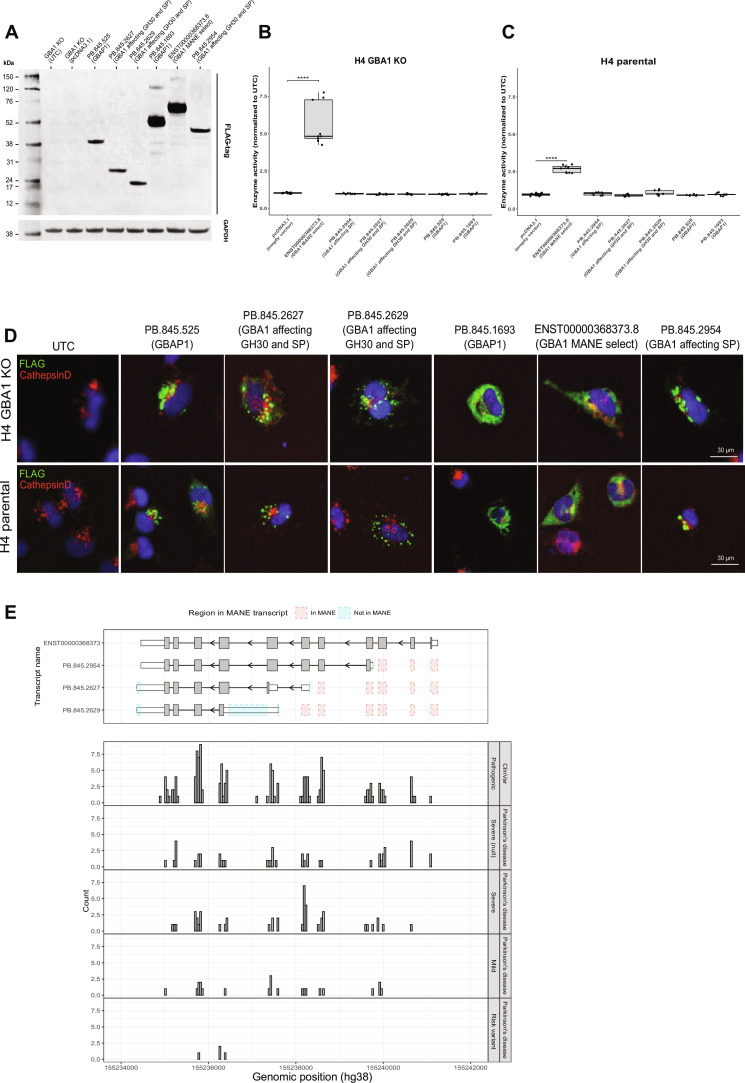
Novel *GBA1* and *GBAP1* transcripts are translated with no GCase activity and impaired lysosomal colocalization with implications for variant interpretation. (**A**) Immunoblot of H4 GBA1(−/−/−) knockout cells transiently transfected with *GBA1* and *GBAP1* constructs containing a C-terminal FLAG-tag. GBA1 and GBAP1 expression was detected using FLAG-tag antibody. GAPDH was used as a loading control. The predicted protein sizes are as follows: PB.845.525 (GBAP1; 321 amino acids; 35 kDa), PB.845.2627 (GBA1 affecting GH30 and SP; 219 amino acids; 24 kDa), PB.845.2629 (GBA1 affecting GH30 and SP; 164 amino acids; 18 kDa), PB.845.1693 (GBAP1; 399 amino acids; 44 kDa), ENST00000368373 (GBA1 MANE select; 537 amino acids; 62 kDa), and PB.845.2954 (GBA1 affecting GH30 and SP; 414 amino acids; 46 kDa). (**B**) Lysosomal enzyme assay of H4 GBA(−/−/−) knockout cells transiently transfected with GBA1 and GBAP1 constructs (**C**) and in H4 parental. GCase enzyme activity was significantly increased only in H4 parental and GBA(−/−/−) knockout cells transiently transfected with the GBA1 full-length construct (ENST00000368373), compared to the empty vector control (*n* = 3). (**D**) Lysosomal colocalization is impaired in novel GBA1 and GBAP1 transcripts. Immunohistochemistry of H4 parental and GBA1(−/−/−) knockout (KO) cells transiently transfected with GBA1 and GBAP1 constructs containing a C-terminal FLAG-tag. Colocalization of GBA1-FLAG and GBAP1-FLAG (green) with CathepsinD (red) was detected using FLAG-tag antibody. (**E**) Pathogenic *GBA1* variants from ClinVar and risk variants from the GBA1-PD browser, which include variants described in PD, annotated onto novel coding *GBA1* transcripts plotted using ggtranscript with differences as compared to MANE select (ENST00000368373) highlighted in blue and red.

To explore the coding potential of *GBA1* and *GBAP1* transcripts with novel ORFs, we used a sequence-based approach along with AlphaFold2 (*44*) (which accurately predicts GBA1 structure; fig. S9). We focused on the most highly expressed *GBA1* (*n* = 3) and *GBAP1* (*n* = 2) ORFs (Fig. 5, A and B). Although protein isoforms of both genes were predicted to have highly similar tertiary structures at the C terminus, we predicted that all protein products would be unlikely to have GCase activity due to the partial/full loss of key enzymatic sites or the absence of the lysosomal targeting sequence (LIMP-2 interface region; Fig. 5, C to H, and fig. S10) (*45*, *46*). To assess the coding potential of these novel *GBA1* and *GBAP1* transcripts, we amplified the ORFs and cloned them into a vector with a C-terminal FLAG-tag. We transfected these vectors into H4 cells with homozygous knockout of *GBA1* and found translation of all transcripts as detected with both an anti-FLAG antibody and an antibody directed to the conserved C terminus (Fig. 6A and fig. S11). However, none of these transcripts encoded protein isoforms with GCase activity, including those transcribed from *GBAP1* (Fig. 6B). We also found no evidence to suggest that these protein isoforms inhibited constitutive GCase activity in H4 parental cells expressing GBA1 (Fig. 6C). Nevertheless, this will require further corroboration by the use of an artificial GCase substrate compatible with live imaging to directly determine GCase activity in the lysosomal compartment [e.g., (*47*, *48*)] and/or the quantification of GCase substrate levels by liquid chromatography–mass spectrometry (LC-MS).

Moreover, immunohistochemical analysis conducted on H4 GBA1 knockout and the H4 parental line, expressing endogenous GBA1, revealed the absence of lysosomal localization for PB.845.525 (GBAP1), PB.845.2627, and PB.845.2629 (both GBA1 isoforms affecting the Glyco_hydro_30 domain + signal peptide). Conversely, some degree of lysosomal localization was observed for PB.845.1693 (GBAP1) and potentially PB.845.2954 (GBA1 isoform affecting signal peptide) (Fig. 6D and fig. S12, A and B).

Noteworthy, only about 20 to 30% of expressed GCase construct distributes to the lysosome, as opposed to approximately 50 to 60% of endogenous GCase (fig. S12C), which may be attributed to artifactual protein trafficking due to tagging and overexpression. To address this caveat and to fully understand distribution of the different forms, one will require approaches based on the use of specific antibodies for probing the putative endogenous proteins and/or LC-MS analysis of organelle-specific proteomes (e.g., LysoIP) to identify specific peptides. With no current availability of these antibodies and no access to this type of organelle-specific datasets, we aimed at interrogating public bulk mass spectrometry dataset of human prefrontal cortex (*49*) and human embryonic stem cell-derived microglia-like cell lines (hMGLs) (*50*). Since novel GBA1 isoforms have no unique sequences that differentiate them, we focused on GBAP1 isoforms. We found proteomic support for GBAP1 (PB.845.1693) within the datasets with a protein *Q* value of <0.01. In particular, we identified the unique amino acid sequence QWALDGAEYR, which is unique to GBAP1 and was not identified when searched within the UniProt human protein reviewed dataset. This shows translation of GBAP1 within the human prefrontal cortex and in hMGLs.

To explore the impact this has on variant interpretation, we conducted an analysis of genetic variants spanning the entire *GBA1* gene, encompassing all variants cataloged in ClinVar and the GBA1-PD browser. We discovered that most pathogenic variants are not present in the first two exons of the MANE select transcript (ENST00000368373), despite these data primarily originating from whole-genome sequencing. However, when they are present in these exons, they lead to a more severe phenotype.

These initial exons encode the signal peptide, which plays a critical role in transporting the protein across the membrane of the rough endoplasmic reticulum. Consequently, when not transcribed, it results in a protein without GCase activity. Consistent with our data, the absence of transcription in these exons is associated with a protein lacking GCase activity. Therefore, variants in these exons appear to be linked to a more pronounced clinical outcome, while those situated in later exons exhibit a broader spectrum of phenotypes, ranging from severe GD to PD risk.

### *GBA1* and *GBAP1* transcripts show cell type selectivity in human brain

**Fig. 7.**
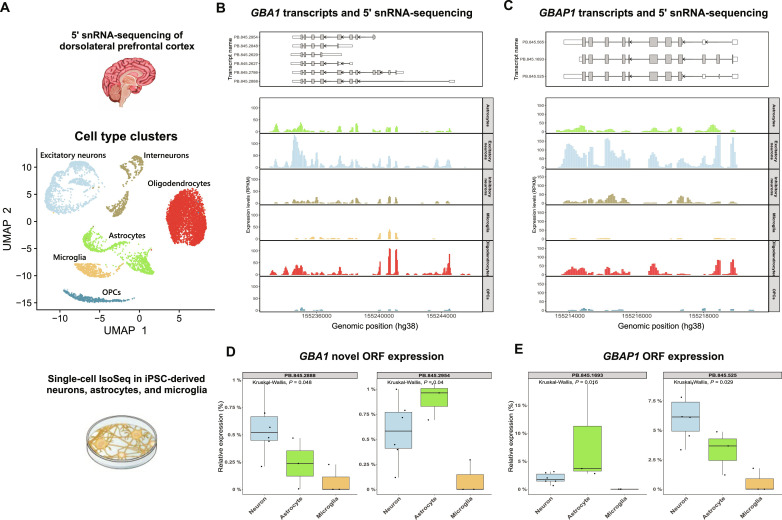
Novel protein coding transcripts of *GBA1* and *GBAP1* show cell type–selective usage. (**A**) Uniform manifold approximation and projection labeled by characterized cell types in human DLPFC. (**B**) *GBA1* expression from 5′ snRNA-seq of human DLPFC. (**C**) *GBAP1* expression from 5′ snRNA-seq of human DLPFC. (**D**) Expression of *GBA1* ORFs from PacBio Iso-Seq data generated from human iPSC-derived cortical neuron (*n* = 6), astrocyte (*n* = 3), and microglia (*n* = 3) cultures. (**E**) Expression of *GBAP1* ORFs from PacBio Iso-Seq data generated from human iPSC-derived cortical neuron (*n* = 6), astrocyte (*n* = 3), and microglia (*n* = 3) cultures.

We found that novel protein-coding transcripts of *GBA1* without predicted GCase activity were common, collectively accounting for between 15.8% (cerebellum) and 31.7% (caudate nucleus) of transcription from the *GBA1* locus. Notably, we found that only 48% of transcription in the caudate nucleus was predicted to encode a protein isoform with GCase activity. This high variability in the usage of *GBA1* transcripts with novel ORFs across the human brain led us to hypothesize that these transcripts may have high cell type specificity. To test this, we used both 5′ single-nucleus RNA-seq (snRNA-seq) of human dorsolateral prefrontal cortex (DLPFC) and targeted PacBio Iso-Seq of human iPSC-derived brain-relevant cell types. Our analysis revealed cell type–selective differences in the expression of *GBA1* and *GBAP1* (Fig. 7).

Specifically, we used 5′ snRNA-seq of DLPFC to assess the expression of *GBA1* and *GBAP1* in various cell types, including astrocytes, excitatory neurons, inhibitory neurons, microglia, oligodendrocytes, and oligodendrocyte precursor cells (OPCs) (Fig. 7A). Our analysis showed an absence of signal at the first exon of PB.845.2888 (*GBA1*) in microglia, along with an overall lower expression of novel *GBA1* transcripts in microglia and OPCs (Fig. 7B). We found that microglia showed significantly lower relative expression of shorter *GBA1* ORFs lacking GCase activity (PB.275.2954 and PB.845.2888) compared to neurons or astrocytes, using PacBio Iso-Seq of human iPSC-derived neurons, astrocytes, and microglia (Fig. 7D).

Likewise, our analysis revealed that excitatory neurons had higher expression of *GBAP1* ORF transcripts as compared to microglia, using 5′ snRNA-seq of DLPFC (Fig. 7C). Further, using PacBio Iso-Seq of human iPSC-derived neurons, astrocytes, and microglia, we found significant cell type–specific differences in *GBAP1* ORF usage, with lower utilization of all *GBAP1* ORFs in microglia compared to excitatory neurons and astrocytes (Fig. 7E). Additionally, our profiling of H3K4me3 mark in neurons using CUT&RUN (*51*) supported transcriptional activity at the 5′ TSSs of *GBAP1* ORF transcripts (fig. S13).

### Inaccurate annotation is frequent among parent genes across human tissues

**Fig. 8.**
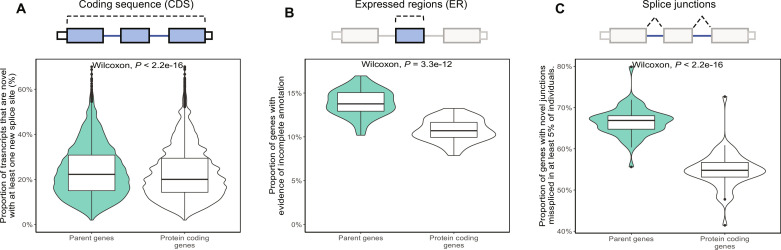
Inaccuracies in annotation are common for parent genes on a genome-wide scale. (**A**) Proportion of transcripts per parent gene and per protein-coding gene without a pseudogene with a novel splice site from long-read RNA-seq data of nine frontal cortex samples. (**B**) Proportion of genes with evidence of incomplete annotation based on the identification of novel expressed genomic regions from short-read RNA-seq data. (**C**) Proportion of genes with evidence of incomplete annotation based on the identification of novel splice junctions found in at least 5% of samples from short-read RNA-seq data.

We have shown substantial inaccuracies in annotation of the parent gene *GBA1*. However, we wanted to explore the scope of this problem. To do so, we compared inaccuracies in annotation of all 3665 parent genes compared with other protein-coding genes (including paralogs). Initially, we used public long-read RNA-seq data from 29 samples (*n*, brain = 9, heart = 16, and lung = 6; table S3) to assess the proportion of transcripts per gene, with at least one novel splice site in the CDS that would result in a novel ORF. Despite a low sequencing depth (mean, 2.2 ± 0.9 million full-length reads per sample), we found a significant increase in such events among parent genes compared to other protein-coding genes (parent genes = 23.9 ± 11.5%; protein-coding genes = 22.7 ± 11.4%; two-sided Wilcoxon rank sum test *P* < 0.01; Fig. 8A). We extended this analysis to a greater number of samples (*n* = 7595) and human tissues (*n* = 41, GTEx) using annotation-agnostic short-read RNA-seq analyses to quantify the proportion of parent genes with evidence of novel splicing (Materials and Methods). On the basis of the identification of novel expressed genomic regions (*38*) and novel splice site usage, we found that the proportion of genes with incomplete annotation was significantly higher among parent genes compared to other protein-coding genes (novel expression regions: parent genes = 13.9 ± 1.4%; protein-coding genes = 10.8 ± 1.3%; two-sided Wilcoxon rank sum test *P* < 0.01; Fig. 8B; splice site usage: parent genes = 66.5 ± 3.5%; protein-coding genes = 54.8 ± 4.3; two-sided Wilcoxon rank sum test *P* < 0.01; Fig. 8C). This observation was consistent across all tissues analyzed (fig. S14).

## DISCUSSION

Here, we show that widespread expression and alternative splicing of pseudogenes in human tissues has limited our understanding of both pseudogene and parent gene transcription with a substantial impact on our appreciation of gene function. Our long-read RNA-seq analysis of the parent gene *GBA1* and its pseudogene *GBAP1* demonstrated notable diversity in transcription and showed that, contrary to expectation (*40*, *41*), no single transcript dominated expression of either gene in human brain. This analysis involved sequencing of polyA-selected RNA and subsequent QC to mitigate the possibility of nascent RNA inclusion. A substantial portion of transcription from both loci was novel, leading to the identification of novel protein-coding transcripts with tissue- and cell type–specific biases in usage. Together, these findings have a substantial impact on our understanding of the potential mechanisms through which genetic variation at the *GBA1-GBAP1* locus could explain phenotypic diversity in GD and modulate disease risk and expressivity in PD.

Although current annotation is known to be incomplete, especially in the brain (*38*), the extent of transcriptional variety and novelty at parent gene loci was surprising, and particularly so at *GBA1*. After all, GCase dysfunction has been implicated in human disease since 1965 (*6*) and mutations in *GBA1* have been described since 1987 (*11*), making *GBA1* one of the most studied genes in the genome. Nonetheless, we found that as much as 31.7% of *GBA1* transcription in the caudate nucleus may be translated into novel protein isoforms that do not localize in lysosomes and, consequently, lack GCase activity. PD is primarily characterized by the degeneration of dopaminergic neurons in the substantia nigra pars compacta (SNpc). SNpc projects dopamine to the striatum, encompassing the caudate nucleus, which, together with the putamen, constitutes a pivotal part of the basal ganglia. Dysfunction in basal ganglia circuitry is a notable feature of PD, and the connection between reduced GCase activity and PD (*13*–*16*) makes these findings even more noteworthy. Moreover, this has implications for variant interpretation. We found that most pathogenic, and risk, variability at *GBA1* would also be translated on novel protein isoforms that do not localize in lysosomes and, thus, lack GCase activity. However, those variants that affect the signal peptide, needed for lysosomal localization and GCase function, seemingly cause a more severe disease. Thus, understanding the specific transcript usage, within disease-relevant tissues and how that relate to GCase activity, might also help in understanding divergent phenotype-genotype relationships for both GD and PD.

While most analyses have focused on *GBA1*-*GBAP1*, we also demonstrate that inaccuracies in annotation were significantly more common across parent genes as compared to other protein-coding genes (Fig. 8) and were not restricted to example. High sequence similarity within the genome and subsequent multimapping of short RNA-seq reads have affected our understanding of many genes, including those already causally linked to disease. Such loci are predictable using sequence similarity analyses, the technology to resolve these “problem” loci is available, and the impact on our understanding of disease is likely to be significant. As exemplified by *GBA1*-*GBAP1*, our limited understanding of transcription from this locus results in errors in quantification of gene expression and all dependent analysis from differential gene expression in disease to quantitative trait loci detection. Beyond a research setting, inaccuracies in annotation will affect variant interpretation and consequently diagnostic yield for some disease-associated genes. Finally, and perhaps most importantly, inaccuracies in transcript annotation impact on our understanding of gene function. Directed by our long-read RNA-seq results, we have found that some *GBAP1* transcripts are more highly expressed in neurons and astrocytes, share a similar predicted three-dimensional (3D) protein structure to GBA1, have protein products that do not localize to lysosomes, and lack GCase activity. Yet, we find robust evidence of translation of such *GBAP1* transcripts in human brain using high-throughput mass spectrometry data (*49*). Extrapolating these findings to GBA1, where mass spectrometry data were uninformative, would suggest a nonlysosomal function for both GBA1 and GBAP1 in brain and particularly in neurons.

We propose that improving our understanding of the molecular functions of parent-pseudogene pairs will become increasingly important to the development and success of RNA-targeting therapies. Accurate annotation is required at the tissue and cell level to design effective antisense oligonucleotides (ASOs) or gene therapies. Furthermore, some pseudogenes may represent particularly high-value therapeutic targets due to their potential to operate as genetic modifiers of Mendelian disorders. Nusinersen, which targets the splicing of former pseudogene *SMN2*, is a highly successful treatment for spinal muscular atrophy (*52*). Thus, a deeper understanding of pseudogene function could lead to innovative therapeutic strategies.

Our results suggest that novel GBA1 isoforms, particularly those lacking GCase activity, may contribute to phenotypic diversity in GD and PD. Further experimentation using top-down proteomics to accurately detect and quantify these novel isoforms and molecular biology techniques to investigate their subcellular localization and functional properties would be necessary to validate this hypothesis. Additionally, our results raise the possibility that novel *GBA1* transcripts, particularly those lacking GCase activity, may have alternative functions. Further experimentation would be required to definitively establish the functional roles of these novel *GBA1* transcripts, but these findings suggest that *GBA1* may have a broader range of functions than previously appreciated.

Together, our findings from the *GBA1-GBAP1* study demonstrate the need for thorough reexamination of transcription in duplicated genomic regions, such as parent-pseudogene pairs. By using accurate full-length transcript sequencing, we are able to resolve these complex loci with unprecedented detail, leading to novel transcript discovery and, as a result, new insights into the functionality of human diseases.

## MATERIALS AND METHODS

### Pseudogenes and parental genes

#### Pseudogene and parent gene annotations

Pseudogene annotations were obtained from GENCODE v38 (https://ftp.ebi.ac.uk/pub/databases/gencode/Gencode_human/) (*25*). We included all HAVANA annotated pseudogenes excluding polymorphic pseudogenes. Biotypes were clustered using the “gene_type” column so that “IG_V_pseudogene,” “IG_C_pseudogene,” “IG_J_pseudogene,” “IG_pseudogene,” TR,” “TR_J_pseudogene,” “TR_V_pseudogene,” “transcribed_unitary_pseudogene,” “unitary_pseudogene” = “Unitary”; “rRNA_pseudogene,” “pseudogene” = “Other”; “transcribed_unprocessed_pseudogene,” “unprocessed_pseudogene,” “translated_unprocessed_pseudogene” = “Unprocessed”; “processed_pseudogene,” “transcribed_processed_pseudo-gene,” “translated_processed_pseudogene” = “Processed.” Parent genes have previously been inferred (*26*) and were obtained from psiCube (http://pseudogene.org/psicube/index.html).

#### Expression analysis from GTEx

Pseudogene and parent gene expression was assessed using median transcript per million (TPM) expression per tissue generated by the GTEx Consortium (v8, accessed on 10 November 2021). As GTEx only uses uniquely mapped reads for expression and multimapping was a concern, expression was assessed as a binary variable. That is, a gene with a median TPM > 0 was considered to be expressed.

For quantitative expression of *GBA1* and *GBAP1*, we used RNA-seq data for 17,510 human samples originating from 54 different human tissues (GTEx, v8) that were downloaded using the R package recount (v1.4.6) (*53*). Cell lines, sex-specific tissues, and tissues with 10 samples or below were removed. Samples with large chromosomal deletions and duplications or large copy number variation previously associated with disease were filtered out (smafrze ! = “EXCLUDE”). For any log_2_ fold change calculations, *GBA1* is the numerator and *GBAP1* is the denominator.

#### Alternative splicing analysis using long-read RNA-seq

To identify alternative splicing of pseudogenes, we used publicly available long-read RNA-seq data from ENCODE (https://www.encodeproject.org/rna-seq/long-read-rna-seq/) (*54*). We included 29 samples from brain (*n* = 9), heart (*n* = 16), and lung (*n* = 6). A description of the samples included can be found in table S2. All samples were sequenced on the PacBio Sequel II platform and processed with the ENCODE DCC deployment of the TALON pipeline (v2.0.0; https://github.com/ENCODE-DCC/long-read-rna-pipeline) (*55*).

#### OMIM data

Phenotype relationships and clinical synopses of all OMIM genes were downloaded using application programming interface through https://omim.org/api (accessed 14 April 2022) (*27*). Parent genes were annotated genes as OMIM morbid if they were listed as causing a Mendelian phenotype.

#### Sequence similarity

Sequence similarity of parent genes and pseudogenes has previously been calculated by Pei *et al.* (*2*) and is available through The Pseudogene Decoration Resource (psiDr; http://www.pseudogene.org/psidr/similarity.dat; accessed 14 April 2022). We compared the sequence similarity of parent and pseudogenes considering the CDS of parent genes.

#### Multimapping from short-read RNA-seq

Multimapping rates of parent genes, including *GBA1* and *GBAP1*, were investigated in human anterior cingulate cortex samples previously reported by Feleke *et al.* (*35*). Here, we used control individuals (*n* = 5) and individuals with PD with or without dementia (*n* = 13). Adapter trimming and read quality filtering was performed with default options using Fastp (v0.23.2; RRID:SCR_016962) (*56*), with QC metrics generated using both Fastp and FastQC (v0.11.9; RRID:SCR_014583). Alignment to the GRCh38 genome using GENCODE v38 was performed using STAR (v2.7.10; RRID:SCR_004463) (*57*). ENCODE standard options for long RNA-seq were used with STAR, except for alignSJDBoverhangMin, outSAMmultNmax, and outFilterMultimapNmax. outFilterMultimapNmax sets the rate of multimapping permitted; as a conservative estimate, we set this to 10, half the ENCODE standard. outSAMmultNmax was set to −1, which allowed multimapped reads to be kept in the same output SAM/BAM file. The QC and alignment processes were performed using a nextflow (*58*) pipeline. BAM files were sorted and indexed using Samtools (v1.14; RRID:SCR_002105) (*59*) and filtered in R (v4.0.5; RRID:SCR_001905) for reads overlapping the *GBA1* or *GBAP1* locus, using GenomicRanges (v1.42.0; RRID:SCR_000025) (*60*) and Rsamtools (version 2.6.0). Only paired first mate reads on the correct strand (minus for both *GBA1* and *GBAP1*) were selected. The “NH” tag, which provides the number of alignments for a read, was also extracted from the SAM header. The CIGAR string of the read was used to provide a width of the reads relative to the reference by adding operations that consume the reference together. Reads were then filtered, using dplyr (v1.0.9; RRID:SCR_016708)(*61*) and tibble (v3.1.6) (*61*), with this new width to leave reads that aligned completely within the *GBA1* and *GBAP1* loci. Reads were then split between unique alignment and multimapping alignments based on the NH tag. The percentage of reads [uniquely mapped/(uniquely mapped + multimapped)] that mapped uniquely to either the *GBA1* or *GBAP1* locus was then calculated. Additionally, for reads that multimapped to the *GBA1* or *GBAP1* locus, the read name was extracted and searched for within the reads that multimapped to the alternate locus (i.e., reads names from reads that multimapped to the *GBA1* locus were searched against read names for reads that multimapped to the *GBAP1* locus). This provided a percentage of reads that aligned to *GBA1* that also aligned elsewhere and the percentage of reads aligning to *GBAP1*. Code and commentary can be found here: https://github.com/Jbrenton191/GBA_multimapping_2022.

### Oxford Nanopore direct cDNA sequencing

#### Samples

Human poly A+ RNA of healthy individuals that passed away from sudden death/trauma derived from frontal lobe and hippocampus was commercially purchased through Clontech (table S2).

#### Direct cDNA sequencing

A total of 100 ng of poly A+ RNA per sample was used for initial cDNA synthesis and subsequent library preparation according to the direct cDNA sequencing (SQK-DCS109) protocol described in detail at protocols.io (dx.doi.org/10.17504/protocols.io.yxmvmkpxng3p/v1). Sequencing was performed on the PromethION using one R9.4.1 flow cell per sample and base-called using Guppy (v4.0.11; ONT, Oxford, UK). Resulting fastq files were processed through a Snakemake pipeline “pipeline-isoforms-ONT-stringtie” [https://github.com/egustavsson/pipeline-ref-isoforms-ONT.git (DOI: https://doi.org/10.5281/zenodo.11091676)]. Gene abundances were calculated implementing the -A parameter in StringTie (v2.2.2 RRID:SCR_016323) (*62*). Data are available and deposited in the Gene Expression Omnibus under accession GSE215459.

#### Comparing short-read quantification versus long-read quantification

For each sample in GTEx, a log_2_ fold change was calculated with *GBA1* as the numerator and *GBAP1* as the denominator across frontal lobe and hippocampus. Shapiro-Wilk normality test in each tissue was used to confirm a normal distribution. To compare against ONT long-read quantification, we used Grubbs’ test (maximum normalized residual test) for a single outlier.

### PACBIO targeted Iso-Seq

#### Samples

Human brain samples: Human poly A+ RNA of healthy individuals that passed away from sudden death/trauma derived from caudate nucleus, cerebellum, cerebral cortex, corpus callosum, dorsal root ganglion, frontal lobe, hippocampus, medulla oblongata, pons, spinal cord, temporal lobe, and thalamus was commercially purchased through Clontech (table S2).

iPSC, neuroepithelial, neural progenitor, cortical neuron, astrocyte, and microglia cells: Control iPSCs consisted of the previously characterized lines Ctrl1 (*63*), ND41866 (Coriel), RBi001 (EBiSC/Sigma-Aldrich), and SIGi1001 (EBiSC/Sigma-Aldrich) as well as the isogenic line previously generated (*64*). Reagents were purchased from Thermo Fisher Scientific unless otherwise stated. iPSC lines were grown in Essential 8 medium on Geltrex substrate and passaged using 0.5 M EDTA. Cortical neurons were differentiated using dual SMAD inhibition for 10 days (10 μM SB431542 and 1 μM dorsomorphin, Tocris) in N2B27 medium before maturation in N2B27 alone (*65*). Day 100 ± 5 days was taken as the final time point. Astrocytes were generated following a similar neural induction protocol until day 80 before repeatedly passaging cortical neuronal inductions in fibroblast growth factor 2 (FGF2) (10 ng/ml, PeproTech) to enrich for astrocyte precursors. At day 150, to generate mature astrocytes, a 2-week maturation consisted of bone morphogenetic protein 4 (BMP4) (10 ng/ml, Thermo Fisher Scientific) and leukemia inhibitory factor (LIF) (10 ng/ml, Sigma-Aldrich) (*66*). To induce inflammatory conditions, astrocytes were stimulated with tumor necrosis factor–α (TNF-α) (30 ng/ml, PeproTech), interleukin-1α (IL-1α) (3 ng/ml, PeproTech), and C1q (400 ng/ml, Merck) (*67*). iPSC-microglia were differentiated following the protocol of Xiang *et al*. (*68*). Embryoid bodies were generated using 10,000 iPSCs, and myeloid differentiation was initiated in Lonza X-VIVO 15 medium, IL-3 (25 ng/ml, PeproTech), and macrophage colony-stimulating factor (MCSF) (100 ng/ml, PeproTech). Microglia released from embryoid bodies were harvested weekly from 4 weeks and matured in Dulbecco’s modified Eagle’s medium (DMEM)–F12 supplemented with 2% insulin/transferrin/selenium, 1% N2 supplement, 1× GlutaMAX, 1× nonessential amino acids, and insulin (5 ng/ml) supplemented with IL-34 (100 ng/ml, PeproTech), MCSF (25 ng/ml, PeproTech), and transforming growth factor–β1 (TGF-β1) (5 ng/ml, PeproTech). A final 2-day maturation consisted of CXC3L1 (100 ng/ml, PeproTech) and CD200 (100 ng/ml, 2B Scientific). Inflammation was stimulated with lipopolysaccharide (10 ng/ml, Sigma-Aldrich). Total RNA was extracted using the Qiagen RNeasy kit according to the manufacturer’s protocol, with β-mercaptoethanol added to buffer RLT and with a deoxyribonuclease (DNase) digestion step included.

#### cDNA synthesis

A total of 250 ng of RNA was used per sample for reverse transcription. Two different cDNA synthesis approaches were used: (i) Human brain cDNA was generated by SMARTer PCR cDNA synthesis (Takara) and (ii) iPSC-derived cell lines were generated using NEBNext Single Cell/Low Input cDNA Synthesis & Amplification Module (New England Biolabs). For both reactions, sample-specific barcoded oligo dT (12 μM) with PacBio 16-mer barcode sequences was added (table S3).

SMARTer PCR cDNA synthesis: First-strand synthesis was performed as per manufacturer instructions, using sample-specific barcoded primers instead of the 3′ SMART CDS Primer II A. We used a 90-min incubation to generate full-length cDNAs. cDNA amplification was performed using a single primer (5′ PCR Primer II A from the SMARTer kit, 5′-AAGCAGTGGTATCAACGCAGAGTAC-3′) and was used for all PCR reactions after reverse transcription. We followed the manufacturer’s protocol with our determined optimal number of 18 cycles for amplification; this was used for all samples. We used a 6-min extension time to capture longer cDNA transcripts. PCR products were purified separately with 1× ProNex Beads.

NEBNext single-cell/low-input cDNA synthesis and amplification module: A reaction mix of 5.4 μl of total RNA (250 ng in total), 2 μl of barcoded primer, 1.6 μl of deoxynucleotide triphosphate (25 mM) held at 70°C for 5 min. This reaction mix was then combined with 5 μl of NEBNext Single Cell RT Buffer, 3 μl of nuclease-free H_2_O, and 2 μl of NEBNext Single Cell RT Enzyme Mix. The reverse transcription mix was then placed in a thermocycler at 42°C with the lid at 52°C for 75 min and then held at 4°C. On ice, we added 1 μl of Iso-Seq Express Template Switching Oligo and then placed the reaction mix in a thermocycler at 42°C with the lid at 52°C for 15 min. We then added 30 μl of elution buffer (EB) to the 20-μl Reverse Transcription and Template Switching reaction (for a total of 50 μl), which was then purified with 1× ProNex Beads and eluted in 46 μl of EB. cDNA amplification was performed by combining the eluted Reverse Transcription and Template Switching reaction with 50 μl of NEBNext Single Cell cDNA PCR Master Mix, 2 μl of NEBNext Single Cell cDNA PCR Primer, 2 μl of Iso-Seq Express cDNA PCR Primer, and 0.5 μl of NEBNext Cell Lysis Buffer.

#### cDNA capture using IDT xGen Lockdown probes

We used the xGen Hyb Panel Design Tool (https://eu.idtdna.com/site/order/designtool/index/XGENDESIGN) to design nonoverlapping 120-mer hybridization probes against *GBA1* and *GBAP1*. We removed any overlapping probes with repetitive sequences (repeatmasker) and to reduce the density of probes mapping to intronic regions 0.2, which means 1 probe per 1.2 kb. In the end, our probe pool consisted of 119 probes, of which 54 were targeted toward *GBA1* and 65 were targeted toward *GBAP1*.

We pooled an equal mass of barcoded cDNA for a total of 500 ng per capture reaction. Pooled cDNA was combined with 7.5 μl of Cot DNA in a 1.5-ml LoBind tube. We then added 1.8× of ProNex beads to the cDNA pool with Cot DNA, gently mixed the reaction mix 10 times (using a pipette), and incubated for 10 min at room temperature. After two washes with 200 μl of freshly prepared 80% ethanol, we removed any residual ethanol and immediately added 19 μl of hybridization mix consisting of 9.5 μl of 2× Hybridization Buffer, 3 μl of Hybridization Buffer Enhancer, 1 μl of xGen Asym TSO block (25 nmol), 1 μl of polyT block (25 nmol), and 4.5 μl of 1× xGen Lockdown Probe pool. The PacBio targeted Iso-Seq protocol is described in detail at protocols.io (dx.doi.org/10.17504/protocols.io.n92ld9wy9g5b/v1).

#### Automated analysis of Iso-Seq data using Snakemake

For the analysis of targeted PacBio Iso-Seq data, we created two Snakemake (*69*) (v5.32.2; RRID:SCR_003475) pipelines to analyze targeted long-read RNA-seq robustly and systematically:

APTARS (Analysis of PacBio TARgeted Sequencing; https://github.com/sid-sethi/APTARS): For each SMRT cell, two files were required for processing: (i) a subreads.bam and (ii) a FASTA file with primer sequences, including barcode sequences.

Each sequencing run was processed by ccs (v5.0.0; RRID:SCR_021174; https://ccs.how/), which combines multiple subreads of the same SMRTbell molecule and to produce one highly accurate consensus sequence, also called a HiFi read (≥Q20). We used the following parameters: --minLength 10–maxLength 50000–minPasses 3–minSnr 2.5–maxPoaCoverage 0–minPredictedAccuracy 0.99.

Identification of barcodes, demultiplexing, and removal of primers were then performed using lima (v2.0.0; https://lima.how/) invoking–isoseq–peek-guess.

Isoseq3 (v3.4.0; https://github.com/PacificBiosciences/IsoSeq) was then used to (i) remove polyA tails and (ii) identify and remove concatemers/chimeric reads, with the following parameters refine–require-polya, --log-level DEBUG. This was followed by clustering and polishing with the following parameters using cluster flnc.fofn clustered.bam–verbose–use-qvs.

Reads with predicted accuracy ≥0.99 were aligned to the GRCh38 reference genome using minimap2 (*70*) (v2.17; RRID:SCR_018550) using -ax splice:hq -uf–secondary = no. Samtools (*59*) (RRID:SCR_002105; http://www.htslib.org/) was then used to sort and filter the output SAM for the locus of gene of interest, as defined in config.yml.

We used cDNA_Cupcake (v22.0.0; https://github.com/Magdoll/cDNA_Cupcake) to (i) collapse redundant transcripts, using collapse_isoforms_by_sam.py (--dun-merge-5-shorter) and (ii) obtain read counts per sample, using get_abundance_post_collapse.py followed by demux_isoseq_with_genome.py.

Isoforms detected were characterized and classified using SQANTI3 (*71*) (v4.2; https://github.com/ConesaLab/SQANTI3) in combination with GENCODE (v38) comprehensive gene annotation. An isoform was classified as full splice match (FSM) if it aligned with reference genome with the same splice junctions and contained the same number of exons, incomplete splice match (ISM) if it contained fewer 5′ exons than reference genome, novel in catalog (NIC) if it is a novel isoform containing a combination of known donor or acceptor sites, or novel not in catalog (NNC) if it is a novel isoform with at least one novel donor or acceptor site.

PSQAN (Post Sqanti QC Analysis; https://github.com/sid-sethi/PSQAN): Following transcript characterization from SQANTI3, we applied a set of filtering criteria to remove potential genomic contamination and rare PCR artifacts. We removed an isoform if (i) the percent of genomic “A’s” in the downstream 20-bp window was more than 80% (“perc_A_downstream_TTS” > 80), (ii) one of the junctions was predicted to be template switching artifact (“RTS_stage” = TRUE), or (iii) it was not associated with the gene of interest. Using SQANTI’s output of ORF prediction, NMD prediction, and structural categorization based on comparison with the reference annotation (GENCODE), we grouped the identified isoforms into the following categories: (i) noncoding novel—if predicted to be noncoding and not a full-splice match with the reference; (ii) noncoding known—if predicted to be noncoding and a full-splice match with the reference; (iii) NMD novel—if predicted to be coding and NMD, and not a full-splice match with the reference; (iv) NMD known—if predicted to be coding and NMD, and a full-splice match with the reference; (v) coding novel—if predicted to be coding and not NMD, and not a full-splice match with the reference; (vi) coding known (complete match)—if predicted to be coding and not NMD, and a full-splice and untranslated region match with the reference; and (vii) coding known (alternate 3′/5′ end)—if predicted to be coding and not NMD, and a full-splice match with the reference but with an alternate 3′ end, 5′ end, or both 3′ and 5′ end.

Given a transcript *T* in sample *i* with FLR as the number of full-length reads mapped to the transcript *T*, we calculated the normalized full-length reads (NFLR*_Ti_*) as the percentage of total transcription in the sample

$${\text{NFLR}}_{\mathrm{Ti}}=\frac{{\text{FLR}}_{\mathrm{Ti}}}{\sum_{T=1}^{M}{\text{FLR}}_{\mathrm{Ti}}}\times 100$$

where NFLR*_Ti_* represents the normalized full-length read count of transcript *T* in sample *i*, FLR*_Ti_* is the full-length read count of transcript *T* in sample *i*, and *M* is the total number of transcripts identified to be associated with the gene after filtering. Finally, to summarize the expression of a transcript associated with a gene, we calculated the mean of normalized full-length reads (NFLR*_Ti_*) across all the samples

$${\text{NFLR}}_{T}=\frac{\sum_{i=1}^{N}{\text{NFLR}}_{\mathrm{Ti}}}{N}$$

where NFLR*_T_* represents the mean expression of transcript *T* across all samples and *N* is the total number of samples. To remove low-confidence isoforms arising from artifacts, we only selected isoforms fulfilling the following three criteria: (i) expression of minimum 0.1% of total transcription per sample, i.e., NFLR*_Ti_* ≥ 0.1; (ii) a minimum of 80% of total samples passing the NFLR*_Ti_* threshold; and (3) expression of minimum 0.3% of total transcription across samples, i.e., NFLR*_T_* ≥ 0.3.

#### Visualizations of transcripts

For any visualization of transcript structures, we have recently developed ggtranscript (*72*) (v0.99.03; https://github.com/dzhang32/ggtranscript), an R package that extends the incredibly popular tool ggplot2 (*61*) (v3.3.5 RRID; SCR_014601) for visualizing transcript structure and annotation.

#### CAGE-seq analysis

To assess whether predicted 5′ TSSs of novel transcript were in proximity of CAGE peaks, we used data from the FANTOM5 dataset (*42*, *43*). CAGE is based on “cap trapping”: capturing capped full-length RNAs and sequencing only the first 20 to 30 nucleotides from the 5′-end. CAGE peaks were downloaded from the FANTOM5 project (https://fantom.gsc.riken.jp/5/datafiles/reprocessed/hg38_latest/extra/CAGE_peaks/hg38_liftover+new_CAGE_peaks_phase1and2.bed.gz; accessed 20 May 2022).

### Single-nucleus RNA-seq

#### Nuclei extraction of cortical postmortem tissue

Postmortem brain tissue from control individuals with no known history of neurological or neuropsychiatric symptoms was acquired from the Cambridge Brain Bank (ethical approval from the London-Bloomsbury Research Ethics Committee, REC reference: 16/LO/0508). Brains were bisected in the sagittal plane with one-half flash-frozen and stored at −80°C and the other half fixed in 10% neutral buffered formalin for 2 to 3 weeks. From the flash-frozen blocks, 50 to 100 mg were sampled from the DLPFC (Brodmann area 46) and stored at −80°C until use.

Nuclei were isolated as previously described (*73*), with minor modifications. Approximately 20 μg of −80°C-conserved tissue was thawed and dissociated in ice-cold lysis buffer [0.32 M sucrose, 5 mM CaCl_2_, 3 mM MgAc, 0.1 mM Na_2_EDTA, 10 mM tris-HCl (pH 8.0), 1 mM dithiothreitol (DTT)] using a 1-ml glass dounce tissue grinder (Wheaton). The homogenate was slowly and carefully layered on top of a sucrose layer [1.8 M sucrose, 3 mM MgAc, 10 mM tris-HCl (pH 8.0), 1 mM DTT] in centrifuge tubes to create a gradient and then centrifuged at 15,500 rpm for 2 hours and 15 min. After centrifugation, the supernatant was removed and the pellet was softened for 10 min in 100 μl of nuclear storage buffer [15% sucrose, 10 mM tris-HCl (pH 7.2), 70 mM KCl, 2 mM MgCl_2_] before resuspension in 300 μl of dilution buffer [10 mM tris-HCl (pH 7.2), 70 mM KCl, 2 mM MgCl_2_, Draq7 1:1000]. The suspension was then filtered (70-μm cell strainer) and sorted via fluorescence-activated cell sorting (FACS) (FACSAria III, BD Biosciences) at 4°C at a low flow rate, using a 100-μm nozzle [pipette tips and Eppendorf tubes for transferring nuclei were precoated with 1% bovine serum albumin (BSA)]. Nuclei (8500) were sorted for snRNA-seq and then loaded onto the Chromium Next GEM Single Cell 5′ Kit (10x Genomics, PN-1000263). Sequencing libraries were generated with unique dual indices (TT set A) and pooled for sequencing on NovaSeq 6000 (Illumina) using a 100-cycle kit and 28-10-10-90 reads.

#### snRNA-seq analysis

Raw base calls were demultiplexed to obtain sample-specific FASTQ files using Cell Ranger mkfastq and default parameters (v6; 10x Genomics; RRID:SCR_017344). Reads were aligned to the GRCh38 genome assembly using the Cell Ranger count (v6; 10x Genomics; RRID:SCR_017344) with default parameters (--include-introns were used for nuclei mapping) (*74*). Nuclei were filtered based on the number of genes detected—nuclei with less of the mean minus an SD or more than the mean plus two SDs were discarded to exclude low-quality nuclei or possible doublets. The data were normalized to center log ratio (CLR) to reduce sequencing depth variability. Clusters were defined with Seurat function FindClusters (v4.1.1; RRID:SCR_007322) using resolution of 0.5. Obtained clusters were manually annotated using canonical marker gene expression (table S5).

#### Signal of GBA1/GBAP1 per cell type

Barcodes (grouped by sample and cell type) were used to create Cluster objects from the python package trusTEr (v0.1.1; https://github.com/raquelgarza/truster) and processed with the following functions:

1) tsv_to_bam()—extracts the given barcodes from a sample’s BAM file (outs/possorted_genome_bam.bam output from Cell Ranger count) using the subset-bam software from 10x Genomics (v1.0). Outputs one BAM file for each cell type per sample, which contains all alignments.

2) filter_UMIs()—filters BAM files to only keep unique combinations of cell barcodes, unique molecular identifier (UMI), and sequences.

3) bam_to_fastq()—uses bamtofastq from 10x Genomics (version 1.2.0) to output the filtered BAM files as fastQ files.

4) concatenate_lanes()—concatenates the different lanes (as output from bamtofastq) from one library and generates one FASTQ file per cluster.

5) merge_clusters()—concatenates the resulting FASTQ files (one for each cell type and sample) in defined groups of samples. Here, groups were set to PD or control depending on the diagnosis of the individual from which the sample was derived. Output is a FASTQ file per cell type per condition.

6) map_clusters()—the resulting FASTQ files were then mapped using STAR (v2.7.8a). Multimapping reads were allowed to map up to 100 loci (outFilterMultimapNmax 100, winAnchorMultimapNmax 200); the rest of the parameters were used as default.

The resulting BAM files were converted to bigwig files using bamCoverage and normalized by the number of nuclei per group (expression was multiplied by a scale factor of 1 × 10^7^ and divided by the number of nuclei in a particular cell type) (deeptools v2.5.4; RRID:SCR_016366).

For more details, please refer to the scripts process_celltypes_control_PFCTX.py, celltypes_characterization_PFCTX_Ctl.Rmd, and Snakefile_celltypes_control_PFCTX at GitHub (https://github.com/raquelgarza/GBA_snRNAseq_cutnrun_Gustavsson2022.git).

### CUT&RUN

Postmortem brain tissue from control individuals with no known history of neurological or neuropsychiatric symptoms was acquired from the Skåne University Hospital Tissue Bank (ethical approvement Ethical Committee in Lund, 06582-2019 and 00080-2019). From the flash-frozen tissue, 50 to 100 mg were sampled from the DLPFC and stored at −80°C until use.

CUT&RUN was performed as previously described (*75*), with minor modifications. Concanavalin A (ConA)-coated magnetic beads (Epicypher) were activated by washing twice in bead binding buffer [20 mM Hepes (pH 7.5), 10 mM KCl, 1 mM CaCl, 1 mM MnCl_2_] and placed on ice until use. For adult neuronal samples, nuclei were isolated from frozen tissue as described above (see the “Nuclei extraction of cortical postmortem tissue” section). Before FACS, nuclei were incubated with Recombinant Alexa Fluor 488 Anti-NeuN antibody [EPR12763] - Neuronal Marker (ab190195) at a concentration of 1:500 for 30 min on ice. The nuclei were run through the FACS at 4°C at a low flow rate using a 100-μm nozzle. Alexa Fluor 488–positive nuclei (300,000) were sorted. The sorted nuclei were pelleted at 1300*g* for 15 min and resuspended in 1 ml of ice-cold nuclear wash buffer (20 mM Hepes, 150 mM NaCl, 0.5 mM spermidine, 1× cOmplete protease inhibitors, 0.1% BSA). Thirty microliters (10 μl per antibody treatment) of ConA-coated magnetic beads (Epicypher) were added during gentle vortexing (pipette tips for transferring nuclei were precoated with 1% BSA). Binding of nuclei to beads proceeded for 10 min at room temperature with gentle rotation, and then bead-bound nuclei were split into equal volumes [corresponding to immunoglobulin G (IgG) control and H3K4me3 treatments]. After removal of the wash buffer, nuclei were then resuspended in 100 μl of cold nuclear antibody buffer [20 mM Hepes (pH 7.5), 0.15 M NaCl, 0.5 mM Spermidine, 1× Roche complete protease inhibitors, 0.02% (w/v) digitonin, 0.1% BSA, 2 mM EDTA] containing primary antibody (rabbit anti-H3K4me3 Active Motif 39159, RRID:AB_2615077, or goat anti-rabbit IgG, Abcam ab97047, RRID:AB_10681025) at 1:50 dilution and incubated at 4°C overnight with gentle shaking. Nuclei were washed thoroughly with nuclear digitonin wash buffer [20 mM Hepes (pH 7.5), 150 mM NaCl, 0.5 mM spermidine, 1× Roche cOmplete protease inhibitors, 0.02% digitonin, 0.1% BSA] on the magnetic stand. After the final wash, Protein A and Micrococcal Nuclease (pA-MNase) (a gift from S. Henikoff) was added in nuclear digitonin wash buffer and incubated with the nuclei at 4°C for 1 hour. Nuclei were washed twice, resuspended in 100 μl of digitonin buffer, and chilled to 0° to 2°C in a metal block sitting in wet ice. Genome cleavage was stimulated by addition of 2 mM CaCl_2_ at 0°C for 30 min. The reaction was quenched by addition of 100 μl of 2× stop buffer [0.35 M NaCl, 20 mM EDTA, 4 mM EGTA, 0.02% digitonin, glycogen (50 ng/μl), ribonuclease A (50 ng/μl), yeast spike-in DNA (10 fg/μl) (a gift from S. Henikoff)] and vortexing. After 30-min incubation at 37°C to release genomic fragments, bead-bound nuclei were placed on the magnet stand and fragments from the supernatant were purified by a NucleoSpin clean-up kit (Macherey-Bagel). Illumina sequencing libraries were prepared using the Hyperprep kit (KAPA) with unique dual-indexed adapters (KAPA), pooled, and sequenced on a NextSeq 500 instrument (Illumina).

#### CUT&RUN analysis

Paired-end reads (2 × 150 bp) were aligned to the hg38 genome using bowtie2 (*76*) (v2.3.4.2; RRID:SCR_016368) (--local–very-sensitive-local–no-mixed–no-discordant–phred33 -I 10 -X 700), converted to bam files with samtools (*59*) (v1.4; RRID:SCR_002105), and indexed with samtools (*59*) (v1.9; RRID:SCR_002105). Normalized bigwig coverage tracks were made with bamCoverage (deepTools (*77*) v2.5.4; RRID:SCR_016366), with reads per kilobase of exon per million reads mapped normalization. For more details, please refer to the pipeline Snakefile_Neun_cutnrun in GitHub (https://github.com/raquelgarza/GBA_snRNAseq_cutnrun_Gustavsson2022.git).

### Translation of novel transcripts

#### Structure predictions

Protein sequences of the different isoforms were aligned pairwise to MANE select with BioPython using a BLOSUM62 scoring matrix with gap open penalty of −3 and gap extend penalty of −0.1. pLDDT scores for residues from AlphaFold2 models were extracted and mapped onto the sequence of MANE select according to the alignment. While the structure of the predictions of newly detected isoforms follows mostly the known GBA1 structure, a noteworthy breakdown of the confidence score in regions with deletions is visible. This might indicate a conflict between coevolution information and structural templates from dominant isoforms versus the learned physicochemical properties of protein structures, which might be unfavorable in those regions.

#### Cell culture

H4 cells (American Type Culture Collection HTB-148148) with homozygous knockout of GBA1 (ENSG00000177628) were generated using indel-based CRISPR/Cas9 technology [gRNA 5′-TCCATTGGTCTTGAGCCAAG-3′ (reverse orientation) targeting exon 7] via Horizon Discovery Ltd. Cells were cultured in DMEM supplemented with 10% fetal bovine serum at 37°C, 5% CO_2_. Cells were subcultured every 3 to 4 days at a split ratio of 1:6.

#### Cell transfection

Cells were transfected using Lipofectamine 3000 reagent (Invitrogen L3000008) according to the manufacturer’s instructions. *GBA1* or *GBAP1* transcripts subcloned in the pcDNA3.1(+)-C-DYK vector were designed using the GenSmart design tool and acquired from GenScript.

#### Western blot

Protein was extracted from whole cells using MSD lysis buffer (MSD R60TX-3) containing 1× cOmplete Mini Protease Inhibitor Cocktail (Roche 11836153001) and 1× PhosSTOP Phosphatase Inhibitor Cocktail (Roche 4906845001). Protein concentration was determined by bicinchoninic acid (BCA) assay according to the manufacturer’s instructions (Pierce 23225). Protein (10 to 20 μg) was diluted in NuPAGE LDS Sample Buffer (Invitrogen NP0007) and 200 mM DTT was loaded on NuPAGE 4 to 12% bis-tris mini protein gels. Gels were run in NuPAGE MES SDS Running Buffer (Invitrogen NP0002) at 150 V and transferred to 0.2-μm nitrocellulose membranes in tris-glycine transfer buffer containing 20% MeOH at 30 V for 1.5 hours. Subsequently, membranes were blocked in Intercept Blocking Buffer (LI-COR 927-60001) and incubated with primary antibodies overnight at 4°C and then IRdye-conjugated secondary antibodies before imaging on the LI-COR Biosciences Odyssey CLx imaging system. Primary antibodies used include mouse anti-FLAG (Sigma-Aldrich F3165), rabbit anti-GBA1 (C-terminal; Sigma-Aldrich G4171), and rabbit anti–glyceraldehyde-3-phosphate dehydrogenase (GAPDH) (Abcam ab9485).

#### GCase activity assay

Cells cultured on a 96-well plate were washed with phosphate-buffered saline (PBS) (no Ca^2+^, no Mg^2+^) and harvested in activity assay buffer containing 50 mM citric acid/potassium phosphate (pH 5.0 to 5.4), 0.25% (v/v) Triton X-100, 1% (w/v) sodium taurocholate, and 1 mM EDTA. After a cycle of freeze/thaw and 30-min incubation on ice, samples were centrifuged at 3500 rpm for 5 min in 4°C. Supernatant was collected and incubated in 1% BSA and 2 mM 4-methylumbelliferyl-β-d-galactopyranoside (4-MUG; Sigma-Aldrich M3633) for 90 min at 37°C. The reaction was stopped by addition of 1 M glycine (pH 12.5), and fluorescence (excitation, 365 nm; emission, 445 nm) was measured using a SpectraMax M2 microplate reader (Molecular Devices). Enzyme activity was normalized to untransfected controls.

#### Immunofluorescence

Cells cultured on a 96-well plate were fixed in 4% paraformaldehyde for 10 min and methanol for 10 min and permeabilized in 0.3% Triton X-100 for 10 min at room temperature. Cells were then blocked in BlockAce blocking reagent (Bio-Rad BUF029) for 60 min and then incubated with primary antibodies at 4°C overnight. Following washing with PBS with 0.1% Tween 20, cells were incubated with Alexa Fluor secondary antibodies and Hoechst nucleic acid stain. Imaging was performed on the Thunder imager (Leica) and Opera Phenix High-content Screening System (PerkinElmer). The proportion of FLAG-tag staining (representing overexpressed GBA1) that localized to lysosomes was quantified using Harmony High-Content Imaging and Analysis Software (PerkinElmer). For each condition, >100 cells were assessed across two individual wells with nine fields of images taken per well. Primary antibodies used include mouse anti-FLAG (Sigma-Aldrich F3165), mouse anti-GBA1 (Abcam ab55080), and rabbit anti–cathepsin D (Abcam ab75852).

#### Variant interpretation

We retrieved all genetic variants overlapping the *GBA1* locus from ClinVar, using this script https://github.com/egustavsson/long-read_scripts/blob/main/scripts/getClinVarForLoci.sh and subsequently filtered for only pathogenic variants. Since *GBA1* variants associated with risk of PD are not necessarily classified as pathogenic, we also included data from the GBA1-PD browser (https://pdgenetics.shinyapps.io/gba1browser/) (*78*), a manual curation of PD risk variants in *GBA1*.

#### Mass spectrometric analysis of prefrontal cortex proteomes

Public mass spectrometry dataset was retrieved from ProteomeXchange (PXD026370) and from MassIVE (MSV000085698). PXD026370 consisting of human brain tissue was collected postmortem from patients diagnosed with multiple system atrophy (*n* = 45) and from controls (*n* = 30) to perform a comparative quantitative proteome profiling of tissue from the prefrontal cortex (Broadman area 9) (*49*). MSV000085698 consists of label-free mass spectrometry analysis of hMGLs (*50*) [NO_PRINTED_FORM].

The data analysis was performed using MetaMorpheus (v0.0.320; https://github.com/smith-chem-wisc/MetaMorpheus) (*79*). The search was conducted for two GBAP1 isoforms (PB.845.1693 and PB.845.525), and a list of 267 frequent protein contaminants was found within mass spectrometry data as provided by MetaMorpheus. An FDR (false discovery rate) of 1% was applied for presentation of PSMs (peptide spectrum matches), peptides, and proteins following review of decoy target sequences.

The following search settings were used: protease = trypsin; maximum missed cleavages = 2; minimum peptide length = 7; maximum peptide length = unspecified; initiator methionine behavior = Variable; fixed modifications = Carbamidomethyl on C, Carbamidomethyl on U; variable modifications = Oxidation on M; max mods per peptide = 2; max modification isoforms = 1024; precursor mass tolerance = ±5.0000 parts per million (PPM); product mass tolerance = ±20.0000 PPM; report PSM ambiguity = True.

### Annotation of parent genes and protein-coding genes

To explore inaccuracies in annotation of parent genes and protein-coding genes, we applied three independent approaches.

#### Long-read RNA-seq

To identify full-length transcripts with at least one novel splice junction, we used the same long-read RNA-seq samples available from ENCODE (*54*) as previously described. Transcripts with novel splice junction resulting in novel ORF were those transcripts that had a predicted ORF that was not present in GENCODE v38 annotation.

#### Novel expressed regions

Novel unannotated expression (*38*) was downloaded from Visualisation of Expressed Regions (vizER; https://rytenlab.com/browser/app/vizER). The data originate from RNA-seq data in base-level coverage format for 7595 samples originating from 41 different GTEx tissues. Cell lines, sex-specific tissues, and tissues with 10 samples or below were removed. Samples with large chromosomal deletions and duplications or large copy number variation previously associated with disease were filtered out (smafrze = “USE ME”). Coverage for all remaining samples was normalized to a target library size of 40 million 100-bp reads using the area under coverage value provided by recount2 (*53*). For each tissue, base-level coverage was averaged across all samples to calculate the mean base-level coverage. GTEx junction reads, defined as reads with a noncontiguous gapped alignment to the genome, were downloaded using the recount2 resource and filtered to include only junction reads detected in at least 5% of samples for a given tissue and those that had available donor and acceptor splice sequences.

#### Splice junctions

To identify novel junctions with potential evidence of incomplete annotation, we used data provided by IntroVerse (*80*).

IntroVerse is a relational database that comprises exon-exon split-read data on the splicing of human introns (Ensembl v105) across 17,510 human control RNA samples and 54 tissues originally made available by GTEx and processed by the recount3 project (*34*). RNA-seq reads provided by the GTEx v8 project were sequenced using the Illumina TruSeq library construction protocol (nonstranded 76-bp-long reads, polyA+ selection). Samples from GTEx v8 were processed by recount3 through Monorail [STAR (*57*)] to detect and summarize splice junctions and Megadepth (*81*) to analyze the bam files produced by STAR. Additional QC criteria applied by IntroVerse included (i) exclusively analyzing samples passing the GTEx v8 minimum standards (smafrze ! = “EXCLUDE”), (ii) discarding any split reads overlapping any of the sequences included in the ENCODE Blacklist (*82*), or (iii) split reads that presented an implied intron length shorter than 25 bp.

Second, we extracted all novel donor and acceptor junctions that had evidence of use in ≥5% of the samples of each tissue and grouped them by gene. We then classify those genes either as “parent” or “protein-coding.” Finally, we calculated the proportion that each category of genes presented within each tissue. Focusing on the parent genes category, this can be described as follows

$$P_{T}^{j}=\frac{j}{x}$$

Let *j* denote the total number of parent genes containing at least one novel junction shared by ≥5% of the samples of the current tissue. Let *x* denote the total number of parent genes available for study. Let *T* denote the current tissue.

We mirrored the formula above to calculate the proportion of protein-coding genes per tissue.
