# Supplementary material for: The annotation of GBA1 has been concealed by its protein-coding pseudogene GBAP1
Source: Sci Adv. 2024 Jun 26;10(26):eadk1296. doi: 10.1126/sciadv.adk1296 (PMC11204300; doi:10.1126/sciadv.adk1296)
Supplement: Supplementary file 1 — Supplementary Materials and Methods Figs. S1 to S14 Legends for tables S1 to S5 [file sciadv.adk1296_sm.pdf]

Science Advances
AAAS

Supplementary Materials for

The annotation of *GBA1* has been concealed by its protein-coding pseudogene *GBAP1*

Emil K. Gustavsson *et al.*

Corresponding author: Mina Ryten, mina.ryten@ucl.ac.uk

Sci. Adv. **10**, eadk1296 (2024)
DOI: 10.1126/sciadv.adk1296

The PDF file includes:

Supplementary Materials and Methods
Figs. S1 to S14
Legends for tables S1 to S5

Other Supplementary Material for this manuscript includes the following:

Tables S1 to S5

Materials and Methods

PSEUDOGENES AND PARENTAL GENES

Pseudogene and parent gene annotations

Pseudogene annotations were obtained from GENCODE v 38(*24*) (https://ftp.ebi.ac.uk/pub/databases/gencode/Gencode_human/). We included all HAVANA annotated pseudogenes excluding polymorphic pseudogenes. Biotypes were clustered using the "gene_type" column so that "IG_V_pseudogene", "IG_C_pseudogene", "IG_J_pseudogene", "IG_pseudogene", "TR", "TR_J_pseudogene", "TR_V_pseudogene", "transcribed_unitary_pseudogene", "unitary_pseudogene" = "Unitary"; "rRNA_pseudogene", "pseudogene" = "Other"; "transcribed_unprocessed_pseudogene", "unprocessed_pseudogene", "translated_unprocessed_pseudogene" = "Unprocessed"; "processed_pseudogene", "transcribed_processed_pseudogene", "translated_processed_pseudogene" = "Processed". Parent genes have previously been inferred(*25*) and were obtained from psiCube (http://pseudogene.org/psicube/index.html).

Expression analysis from GTEx

Pseudogene and parent gene expression was assessed using median transcript per million (TPM) expression per tissue generated by the Genotype-Tissue Expression Consortium (GTEx, v8, accessed on 10/11/2021). As GTEx only use uniquely mapped reads for expression and multimapping was a concern, expression was assessed as a binary variable. That is, a gene with a median TPM > 0 was considered to be expressed.

For quantitative expression of *GBA1* and *GBAP1* we used RNA-seq data for 17,510 human samples originating from 54 different human tissues (GTEx, v8) that was downloaded using the R package recount (v 1.4.6)(*55*). Cell lines, sex-specific tissues, and tissues with 10 samples or below were removed. Samples with large chromosomal deletions and duplications or large copy number variation previously associated with disease were filtered out (smafrze != "EXCLUDE"). For any $\log_2$ fold change calculations *GBA1* is the numerator and *GBAP1* is the denominator.

Alternative splicing analysis using long-read RNA-sequencing

To identify alternative splicing of pseudogenes we used publicly available long-read RNA-seq data from ENCODE(*56*) (https://www.encodeproject.org/rna-seq/long-read-rna-seq/). We

included 29 samples from Brain (n = 9), Heart (n = 16) and lung (n = 6). A description of the samples included can be found in **Supplementary Table 2**. All samples were sequenced on the PacBio Sequel II platform and processed with the ENCODE DCC deployment of the TALON pipeline (v2.0.0; https://github.com/ENCODE-DCC/long-read-rna-pipeline)(*57*).

Online Mendelian Inheritance in Man data

Phenotype relationships and clinical synopses of all Online Mendelian Inheritance in Man (OMIM) genes were downloaded using API through https://omim.org/api (accessed 14/04/2022)(*26*). Parent genes were annotated genes as OMIM morbid if they were listed as causing a mendelian phenotype.

Sequence similarity

Sequence similarity of parent genes and pseudogenes has previously been calculated by Pei *et al*.(*2*) and is available through The Pseudogene Decoration Resource (psiDr; http://www.pseudogene.org/psidr/similarity.dat; accessed 14/04/2022). We compared the sequence similarity of parent and pseudogenes considering the coding sequence (CDS) of parent genes.

Multimapping from short-read RNA-sequencing

Multimapping rates of parent genes, including *GBA1* and *GBAP1*, were investigated in human anterior cingulate cortex samples previously reported in Feleke & Reynolds et al(*34*). Here, we used control individuals (n = 5) and individuals with Parkinson's disease (PD) with or without dementia (n = 13). Adapter trimming and read quality filtering was performed with default options using Fastp (v 0.23.2; RRID:SCR_016962)(*58*), with quality control metrics generated using both Fastp and FastQC (v 0.11.9; RRID:SCR_014583). Alignment to the GRCh38 genome using GENCODE v 38 was performed using STAR (v 2.7.10; RRID:SCR_004463)(*59*). ENCODE standard options for long RNA-seq were used with STAR, except for alignSJDBoverhangMin, outSAMmultNmax and outFilterMultimapNmax. outFilterMultimapNmax sets the rate of multimapping permitted; as a conservative estimate we set this to 10, half the ENCODE standard. outSAMmultNmax was set to -1, which allowed multimapped reads to be kept in the same output SAM/BAM file. The QC and alignment processes were performed using a nextflow(*60*) pipeline. BAM files were sorted and indexed

using Samtools (v 1.14; RRID:SCR_002105) (*61*) and filtered in R (v 4.0.5; RRID:SCR_001905) for reads overlapping the *GBA1* or *GBAP1* locus, using GenomicRanges (v 1.42.0; RRID:SCR_000025)(*62*) and Rsamtools (version 2.6.0). Only paired first mate reads on the correct strand (minus for both *GBA1* and *GBAP1*) selected. The "NH" tag, which provides the number of alignments for a read was also extracted from the SAM header. The CIGAR string of the read was used to provide a width of the reads relative to the reference by adding operations that consume the reference together. Reads were then filtered, using dplyr (v 1.0.9; RRID:SCR_016708)(*63*) and tibble (v 3.1.6)(*63*), with this new width to leave reads that aligned completely within the *GBA1* and *GBAP1* locus. Reads were then split between unique alignment and multimapping alignments based on the "NH" tag. The percentage of reads (uniquely mapped / (uniquely mapped + multimapped)) that mapped uniquely to either the *GBA1* or *GBAP1* locus was then calculated. Additionally, for reads that multimapped to the *GBA1* or *GBAP1* locus the read name was extracted and searched for within the reads that multimapped to the alternate locus (i.e., reads names from reads that multimapped to the *GBA1* locus were searched against read names for reads that multimapped to the *GBAP1* locus). This provided a percentage of reads that aligned to *GBA1* that that also aligned elsewhere and the percentage of reads aligning to *GBAP1*. Code and commentary can be found here: https://github.com/Jbrenton191/GBA_multimapping_2022.

OXFORD NANOPORE DIRECT CDNA SEQUENCING

Samples

Human Poly A+ RNA of healthy individuals that passed away from sudden death/trauma derived from frontal lobe and hippocampus were commercially purchased through Clontech (**Supplementary Table 2**).

Direct cDNA sequencing

A total of 100ng of Poly A+ RNA per sample was used for initial cDNA synthesis and subsequent library preparation according to the direct cDNA sequencing (SQK-DCS109) protocol described in detail at protocols.io (dx.doi.org/10.17504/protocols.io.yxmvmkpxng3p/v1). Sequencing was performed on the PromethION using one R9.4.1 flow cell per sample and base-called using Guppy (v 4.0.11; Oxford Nanopore Technologies—ONT, Oxford, UK). Resulting fastq files were processed

through the "pipeline-nanopore-ref-isoforms" (https://github.com/nanoporetech/pipeline-nanopore-ref-isoforms). Gene abundances was calculated implementing the -A parameter in StringTie (v 2.1.1 RRID:SCR_016323)(*64*). Data is available and deposited in the Gene Expression Omnibus under accession GSE215459

Comparing short-read quantification versus long-read quantification

For each sample in GTEx a log2 fold change was calculated with *GBA1* as the numerator and *GBAP1* as the denominator across frontal lobe and hippocampus. Shapiro-Wilk normality test in each tissue was used to confirm a normal distribution. To compare against ONT long-read quantification we used Grubbs' test (maximum normalized residual test) for a single outlier.

PACBIO TARGETED ISO-SEQ

Samples

Human brain samples: Human Poly A+ RNA of healthy individuals that passed away from sudden death/trauma derived from caudate nucleus, cerebellum, cerebral cortex, corpus callosum, dorsal root ganglion, frontal lobe, hippocampus, medulla oblongata, pons, spinal cord, temporal lobe, and thalamus were commercially purchased through Clontech (**Supplementary Table 2)**.

iPSC, neuroepithelial, neural progenitor, cortical neuron, astrocyte, and microglia cells: Control iPSCs consisted of the previously characterized lines Ctrl1(*65*), ND41866 (Coriel), RBi001 (EBiSC/Sigma) and SIGi1001 (EBiSC/Sigma) as well as the isogenic line previously generated(*66*). Reagents were purchased from Thermo Fisher Scientific unless otherwise stated. iPSCs lines were grown in Essential 8 media on geltrex substrate and passaged using 0.5M EDTA. Cortical neurons were differentiated using dual SMAD inhibition for 10 days (10µM SB431542 and 1µM dorsomorphin, Tocris) in N2B27 media before maturation in N2B27 alone(*67*). Day 100 +/- 5 days was taken as the final timepoint. Astrocytes were generated following a similar neural induction protocol until day 80 before repeatedly passaging cortical neuronal inductions in 10ng/ml FGF2 (Peprotech) to enrich for astrocyte precursors. At day 150, to generate mature astrocytes, a two-week maturation consisted of BMP4 (10ng/ml, Thermo Fisher) and LIF (10ng/ml, Sigma)(*68*). To induce inflammatory conditions, astrocytes were stimulated with TNFα (30ng/ml, Peprotech), IL1α (3ng/ml, Peprotech) and C1q (400ng/ml,

Merck)(*69*). iPSC-microglia were differentiated following the protocol of Xiang at al(*70*). Embryoid bodies were generated using 10,000 iPSCs and myeloid differentiation was initiated in Lonza XVivo 15 media, IL3 (25ng/ml, Peprotech) and MCSF (100ng/ml, Peprotech). Microglia released from embryoid bodies were harvested weekly from 4 weeks and matured in DMEM-F12 supplemented with 2% insulin/transferrin/selenium, 1% N2 supplement, 1X glutamax, 1X NEAA and 5ng/ml insulin supplemented with IL34 (100ng/ml, Peprotech), MCSF (25ng/ml, Peprotech), TGFβ1 (5ng/ml, Peprotech). A final two-day maturation consisted of CXC3L1 (100ng/ml, Peprotech) and CD200 (100ng/ml, 2B Scientific). Inflammation was stimulated with lipopolysaccharide (10ng/ml, Sigma).

Total RNA was extracted using the Qiagen RNeasy kit according to the manufacturer's protocol with β-mercaptoethanol added to buffer RLT and with a DNase digestion step included.

cDNA synthesis

A total of 250ng of RNA was used per sample for reverse transcription. Two different cDNA synthesis approaches were used: (i) Human brain cDNA was generated by SMARTer PCR cDNA synthesis (Takara) and (ii) iPSC derived cell lines were generated using NEBNext® Single Cell/Low Input cDNA Synthesis & Amplification Module (New England Biolabs). For both reactions sample-specific barcoded oligo dT (12 µM) with PacBio 16mer barcode sequences were added (**Supplementary Table 3**).

SMARTer PCR cDNA synthesis: First strand synthesis was performed as per manufacturer instructions, using sample-specific barcoded primers instead of the 3' SMART CDS Primer II A. We used a 90 min incubation to generate full-length cDNAs. cDNA amplification was performed using a single primer (5' PCR Primer II A from the SMARTer kit, 5′ AAG CAG TGG TAT CAA CGC AGA GTA C 3′) and was used for all PCR reactions post reverse transcription. We followed the manufacturer's protocol with our determined optimal number of 18 cycles for amplification; this was used for all samples. We used a 6 min extension time in order to capture longer cDNA transcripts. PCR products were purified separately with 1X ProNex® Beads.

NEBNext® Single Cell/Low Input cDNA Synthesis & Amplification Module: A reaction mix of 5.4 µL of total RNA (250 ng in total), 2 µL of barcoded primer, 1.6 µL of dNTP (25 mM) held at 70°C for 5 min. This reaction mix was then combined with 5 µL of NEBNext Single Cell RT Buffer, 3 µL of nuclease-free H_2O and 2 µL NEBNext Single Cell RT Enzyme Mix. The reverse

transcription mix was then placed in a thermocycler at 42°C with the lid at 52°C for 75 minutes then held at 4°C. On ice, we added 1 µL of Iso-Seq Express Template Switching Oligo and then placed the reaction mix in a thermocycler at 42°C with the lid at 52°C for 15 minutes. We then added 30 µL elution buffer (EB) to the 20 µL Reverse Transcription and Template Switching reaction (for a total of 50 µL), which was then purified with 1X ProNex® Beads and eluted in 46 µL of EB. cDNA amplification was performed by combining the eluted Reverse Transcription and Template Switching reaction with 50 µL of NEBNext Single Cell cDNA PCR Master Mix, 2 µL of NEBNext Single Cell cDNA PCR Primer, 2 µL of Iso-Seq Express cDNA PCR Primer and 0.5 µL of NEBNext Cell Lysis Buffer.

cDNA Capture Using IDT Xgen® Lockdown® Probes

We used the xGen Hyb Panel Design Tool (https://eu.idtdna.com/site/order/designtool/index/XGENDESIGN) to design non-overlapping 120-mer hybridization probes against *GBA1* and *GBAP1*. We removed any overlapping probes with repetitive sequences (repeatmasker) and to reduce the density of probes mapping to intronic regions 0.2, which means 1 probe per 1.2kb. In the end, our probe pool consisted of 119 probes of which 54 were targeted towards *GBA1* and 65 were targeted towards *GBAP1*.

We pooled an equal mass of barcoded cDNA for a total of 500 ng per capture reaction. Pooled cDNA was combined with 7.5 µL of Cot DNA in a 1.5 mL LoBind tube. We then added 1.8X of ProNex beads to the cDNA pool with Cot DNA, gently mixed the reaction mix 10 times (using a pipette) and incubated for 10 min at room temperature. After two washes with 200 µL of freshly prepared 80% ethanol, we removed any residual ethanol and immediately added 19 µL hybridization mix consisting of: 9.5 µL of 2X Hybridization Buffer, 3 µL of Hybridization Buffer Enhancer, 1 µL of xGen Asym TSO block (25 nmole), 1 µL of polyT block (25 nmole) and 4.5 µL of 1X xGen Lockdown Probe pool.

The PacBio targeted Iso-Seq protocol is described in detail at protocols.io (dx.doi.org/10.17504/protocols.io.n92ld9wy9g5b/v1).

Automated Analysis of Iso-Seq data using Snakemake

For the analysis of targeted PacBio Iso-Seq data, we created two Snakemake(*71*) (v 5.32.2; RRID:SCR_003475) pipelines to analyse targeted long-read RNA-seq robustly and systematically:

APTARS (Analysis of PacBio TARgeted Sequencing, https://github.com/sid-sethi/APTARS): For each SMRT cell, two files were required for processing: (i) a subreads.bam and (ii) a FASTA file with primer sequences, including barcode sequences.

Each sequencing run was processed by ccs (v 5.0.0; RRID:SCR_021174; https://ccs.how/), which combines multiple subreads of the same SMRTbell molecule and to produce one highly accurate consensus sequence, also called a HiFi read (≥ Q20). We used the following parameters: --minLength 10 –maxLength 50000 –minPasses 3 –minSnr 2.5 –maxPoaCoverage 0 –minPredictedAccuracy 0.99.

Identification of barcodes, demultiplexing and removal of primers was then performed using lima (v 2.0.0; https://lima.how/) invoking –isoseq –peek-guess.

Isoseq3 (v 3.4.0; https://github.com/PacificBiosciences/IsoSeq) was then used to (i) remove polyA tails and (ii) identify and remove concatemers using, with the following parameters refine –require-polya, --log-level DEBUG. This was followed by clustering and polishing with the following parameters using: cluster flnc.fofn clustered.bam –verbose –use-qvs.

Reads with predicted accuracy ≥ 0.99 were aligned to the GRCh38 reference genome using minimap2(*72*) (v 2.17; RRID:SCR_018550) using -ax splice:hq -uf –secondary=no. samtools(*61*) (RRID:SCR_002105; http://www.htslib.org/) was then used to sort and filter the output SAM for the locus of gene of interest, as defined in the config.yml.

We used cDNA_Cupcake (v 22.0.0; https://github.com/Magdoll/cDNA_Cupcake) to: (i) collapse redundant transcripts, using collapse_isoforms_by_sam.py (--dun-merge-5-shorter) and (ii) obtain read counts per sample, using get_abundance_post_collapse.py followed by demux_isoseq_with_genome.py.

Isoforms detected were characterized and classified using SQANTI3(*73*) (v 4.2; https://github.com/ConesaLab/SQANTI3) in combination with GENCODE (v 38) comprehensive gene annotation. An isoform was classified as full splice match (FSM) if it aligned with reference genome with the same splice junctions and contained the same number of

exons, incomplete splice match (ISM) if it contained fewer $5'$ exons than reference genome, novel in catalog (NIC) if it is a novel isoform containing a combination of known donor or acceptor sites, or novel not in catalog (NNC) if it is a novel isoform with at least one novel donor or acceptor site.

PSQAN (Post Sqanti QC Analysis, https://github.com/sid-sethi/PSQAN) Following transcript characterisation from SQANTI3, we applied a set of filtering criteria to remove potential genomic contamination and rare PCR artifacts. We removed an isoform if: (1) the percent of genomic "A" s in the downstream 20 bp window was more than 80% ("perc_A_downstream_TTS" > 80); (2) one of the junctions was predicted to be template switching artifact ("RTS_stage" = TRUE); or (3) it was not associated with the gene of interest. Using SQANTI's output of ORF prediction, NMD prediction and structural categorisation based on comparison with the reference annotation (GENCODE), we grouped the identified isoforms into the following categories: (1) Non-coding novel – if predicted to be non-coding and not a full-splice match with the reference; (2) Non-coding known – if predicted to be non-coding and a full-splice match with the reference; (3) NMD novel – if predicted to be coding & NMD, and not a full-splice match with the reference; (4) NMD known – if predicted to be coding & NMD, and a full-splice match with the reference; (5) Coding novel – if predicted to be coding & not NMD, and not a full-splice match with the reference; (6) Coding known (complete match) – if predicted to be coding & not NMD, and a full-splice & UTR match with the reference; and (7) Coding known (alternate 3'/5' end) – if predicted to be coding & not NMD, and a full-splice match with the reference but with an alternate 3' end, 5' end or both 3' and 5' end.

Given a transcript T in sample i with FLR as the number of full-length reads mapped to the transcript T, we calculated the normalised full-length reads ($NFLR_{Ti}$) as the percentage of total transcription in the sample:

$$NFLR_{Ti} = \frac{FLR_{Ti}}{\sum_{T=1}^{M} FLR_{Ti}} \times 100$$

where, $NFLR_{Ti}$ represents the normalised full-length read count of transcript T in sample i, FLR_{Ti} is the full-length read count of transcript T in sample i and M is the total number of transcripts identified to be associated with the gene after filtering. Finally, to summarise the expression of a transcript associated with a gene, we calculated the mean of normalised full-length reads ($NFLR_{Ti}$) across all the samples:

$$NFLR_T = \frac{\sum_{i=1}^{N} NFLR_{Ti}}{N}$$

where, $NFLR_T$ represents the mean expression of transcript T across all samples and N is the total number of samples. To remove low-confidence isoforms arising from artefacts, we only selected isoforms fulfilling the following three criteria: (1) expression of minimum 0.1% of total transcription per sample, i.e., $NFLR_{Ti} \geq 0.1$; (2) a minimum of 80% of total samples passing the $NFLR_{Ti}$ threshold; and (3) expression of minimum 0.3% of total transcription across samples, i.e., $NFLR_T \geq 0.3$.

Visualizations of transcripts

For any visualization of transcript structures, we have recently developed ggtranscript(*74*) (v 0.99.03; https://github.com/dzhang32/ggtranscript), a R package that extends the incredibly popular tool ggplot2(*63*) (v 3.3.5 RRID; SCR_014601) for visualizing transcript structure and annotation.

CAGE-seq analysis

To assess whether predicted 5' TSSs of novel transcript were in proximity of Cap Analysis Gene Expression (CAGE) peaks we used data from the FANTOM5 dataset(*41*, *42*). CAGE is based on "cap trapping": capturing capped full-length RNAs and sequencing only the first 20–30 nucleotides from the 5'-end. CAGE peaks were downloaded from the FANTOM5 project (https://fantom.gsc.riken.jp/5/datafiles/reprocessed/hg38_latest/extra/CAGE_peaks/hg38_liftover+new_CAGE_peaks_phase1and2.bed.gz; accessed 20/05/2022).

SINGLE NUCLEAR RNA-SEQUENCING

Nuclei extraction of cortical post-mortem tissue

Post-mortem brain tissue from control individuals with no known history of neurological or neuropsychiatric symptoms was acquired from the Cambridge Brain Bank (ethical approval from the London-Bloomsbury Research Ethics Committee, REC reference:16/LO/0508). Brains were bisected in the sagittal plane with one half flash-frozen and stored at -80 °C and the other half fixed in 10% neutral buffered formalin for 2–3 weeks. From the flash-frozen blocks, 50-100mg were sampled from the dorsolateral prefrontal cortex (Brodmann area 46) and stored at -80 °C until use.

Nuclei were isolated as previously described(*75*), with minor modifications. Approximately 20

µg of -80 °C-conserved tissue was thawed and dissociated in ice-cold lysis buffer (0.32M sucrose, 5 mM CaCl2, 3 mM MgAc, 0.1 mM Na2EDTA, 10 mM Tris-HCl pH 8.0, 1 mM DTT) using a 1 mL glass dounce tissue grinder (Wheaton). The homogenate was slowly and carefully layered on top of a sucrose layer (1.8 M sucrose, 3 mM MgAc, 10 mM Tris-HCl pH 8.0, 1 mM DTT) in centrifuge tubes to create a gradient, and then centrifuged at 15,500 rpm for 2 h 15 min. After centrifugation, the supernatant was removed, and the pellet softened for 10 minutes in 100 µL of nuclear storage buffer (15% sucrose, 10 mM Tris-HCl pH 7.2, 70 mM KCl, 2 mM MgCl2) before resuspension in 300 µL of dilution buffer (10 mM Tris-HCl pH 7.2, 70 mM KCl, 2 mM MgCl2, Draq7 1:1000). The suspension was then filtered (70 µm cell strainer) and sorted via FACS (FACS Aria III, BD Biosciences) at 4 °C at a low flowrate, using a 100 µm nozzle (Pipette tips and Eppendorf tubes for transferring nuclei were pre-coated with 1% BSA). 8,500 nuclei were sorted for single-nucleus RNA-seq and then loaded on to the Chromium Next GEM Single Cell 5' Kit (10x Genomics, PN-1000263). Sequencing libraries were generated with unique dual indices (TT set A) and pooled for sequencing on a NovaSeq 6000 (Illumina) using a 100-cycle kit and 28-10-10-90 reads.

Single nucleus RNA-sequencing analysis

Raw base calls were demultiplexed to obtain sample specific FASTQ files using Cell Ranger mkfastq and default parameters (v 6; 10x Genomics; RRID:SCR_017344). Reads were aligned to the GRCh38 genome assembly using the Cell Ranger count (v 6; 10x Genomics; RRID:SCR_017344) with default parameters (--include-introns were used for nuclei mapping)(*76*). Nuclei were filtered based on the number of genes detected - nuclei with less of the mean minus a standard deviation, or more than the mean plus two standard deviations were discarded to exclude low quality nuclei or possible doublets. The data was normalized to center log ratio (CLR) to reduce sequencing depth variability. Clusters were defined with Seurat function FindClusters (v; RRID:SCR_007322), using resolution of 0.5. Obtained clusters were manually annotated using canonical marker gene expression as following:

**Cell type**	**Markers used**
Excitatory neurons	RBFOX3, GRIN1, HS3ST2
Interneurons	GAD1, GAD2, CALB2, CNR1
Astrocytes	GFAP, AQP4, GJA1, SLC1A3
Oligodendrocytes	PLP1, MOG, MBP
OPC (oligodendrocyte precursor cells)	COL9A1, VCAN, PDGFRA

Microglia	FYB1, P2RY12

Signal of GBA1/GBAP1 per cell type

Barcodes (grouped by sample and cell type) were used to create Cluster objects from the python package trusTEr (version 0.1.1; https://github.com/raquelgarza/truster) and processed with the following functions:

1) tsv_to_bam() – extracts the given barcodes from a sample's BAM file (outs/possorted_genome_bam.bam output from Cell Ranger count) using the subset-bam software from 10x Genomics (v 1.0). Outputs one BAM file for each cell type per sample, which contains all alignments.
2) filter_UMIs() – filters BAM files to only keep unique combinations of cell barcodes, UMI, and sequences.
3) bam_to_fastq() – uses bamtofastq from 10x Genomics (version 1.2.0) to outputs the filtered BAM files as fastQ files.
4) concatenate_lanes() – concatenates the different lanes (as output from bamtofastq) from one library and generates one FASTQ file per cluster.
5) merge_clusters() – concatenates the resulting FASTQ files (one for each cell type and sample) in defined groups of samples. Here, groups were set to PD or Control depending on the diagnosis of the individual from which the sample was derived. Output is a FASTQ file per cell type per condition.
6) map_clusters() – the resulting FASTQ files were then mapped using STAR (v 2.7.8a). Multimapping reads were allowed to map up to 100 loci (outFilterMultimapNmax 100, winAnchorMultimapNmax 200), the rest of the parameters were used as default.

The resulting BAM files were converted to bigwig files using bamCoverage and normalized by the number of nuclei per group (expression was multiplied by a scale factor of 1e+07 and divided by the number of nuclei in a particular cell type) (deeptools v 2.5.4; RRID:SCR_016366).

For more details, please refer to the scripts process_celltypes_control_PFCTX.py, celltypes_characterization_PFCTX_Ctl.Rmd, and Snakefile_celltypes_control_PFCTX at the github https://github.com/raquelgarza/GBA_snRNAseq_cutnrun_Gustavsson2022.git.

CUT&RUN

Post-mortem brain tissue from control individuals with no known history of neurological or neuropsychiatric symptoms was acquired from the Skåne University Hospital Tissue Bank (ethical approvement Ethical Committee in Lund, 06582-2019 & 00080-2019). From the flash-frozen tissue, 50-100 mg were sampled from the dorsolateral prefrontal cortex and stored at -80 °C until use.

CUT&RUN was performed as previously described (*77*), with minor modifications. ConA-coated magnetic beads (Epicypher) were activated by washing twice in bead binding buffer (20 mM HEPES pH 7.5, 10 mM KCl, 1 mM CaCl, 1 mM $MnCl_2$) and placed on ice until use. For adult neuronal samples, nuclei were isolated from frozen tissue as described above (see, "Nuclei extraction of cortical post-mortem tissue"). Prior to FACS, nuclei were incubated with Recombinant Alexa Fluor® 488 Anti-NeuN antibody [EPR12763] - Neuronal Marker (ab190195) at a concentration of 1:500 for 30 minutes on ice. The nuclei were run through the FACS at 4 °C at a low flowrate, using a 100 µm nozzle. 300,000 Alexa Fluor – 488 positive nuclei were sorted. The sorted nuclei were pelleted at 1,300 x g for 15 min and resuspended in 1 mL of ice-cold nuclear wash buffer (20 mM HEPES, 150 mM NaCl, 0.5 mM spermidine, 1x cOmplete protease inhibitors, 0.1% BSA). 30 µL (10 µL per antibody treatment) of ConA-coated magnetic beads (Epicypher) were added during gentle vortexing (pipette tips for transferring nuclei were pre-coated with 1% BSA). Binding of nuclei to beads proceeded for 10 min at room temperature with gentle rotation, and then bead-bound nuclei were split into equal volumes (corresponding to IgG control and H3K4me3 treatments). After removal of the wash buffer, nuclei were then resuspended in 100 µL cold nuclear antibody buffer (20 mM HEPES pH 7.5, 0.15 M NaCl, 0.5 mM Spermidine, 1x Roche complete protease inhibitors, 0.02% w/v digitonin, 0.1% BSA, 2 mM EDTA) containing primary antibody (rabbit anti-H3K4me3 Active Motif 39159, RRID:AB_2615077; or goat anti-rabbit IgG, Abcam ab97047, RRID:AB_10681025) at 1:50 dilution and incubated at 4 ºC overnight with gentle shaking. Nuclei were washed thoroughly with nuclear digitonin wash buffer (20 mM HEPES pH 7.5, 150 mM NaCl, 0.5 mM Spermidine, 1x Roche cOmplete protease inhibitors, 0.02% digitonin, 0.1% BSA) on the magnetic stand. After the final wash, pA-MNase (a generous gift from Steve Henikoff) was added in nuclear digitonin wash buffer and incubated with the nuclei at 4 ºC for 1 h. Nuclei were washed twice, resuspended in 100 µL digitonin buffer, and chilled to 0-2 ºC in a metal block

sitting in wet ice. Genome cleavage was stimulated by addition of 2 mM $CaCl_2$ at 0 ºC for 30 min. The reaction was quenched by addition of 100 µL 2x stop buffer (0.35 M NaCl, 20 mM EDTA, 4 mM EGTA, 0.02% digitonin, 50 ng/µL glycogen, 50 ng/µL RNase A, 10 fg/µL yeast spike-in DNA (a generous gift from Steve Henikoff)) and vortexing. After 30 min incubation at 37 ºC to release genomic fragments, bead-bound nuclei were placed on the magnet stand and fragments from the supernatant purified by a NucleoSpin clean-up kit (Macherey-Bagel). Illumina sequencing libraries were prepared using the Hyperprep kit (KAPA) with unique dual-indexed adapters (KAPA), pooled and sequenced on a Nextseq500 instrument (Illumina).

CUT&RUN analysis

Paired-end reads (2x150 bp) were aligned to the hg38 genome using bowtie2(*78*) (v 2.3.4.2; RRID:SCR_016368) (--local –very-sensitive-local –no-mixed –no-discordant –phred33 -I 10 -X 700), converted to bam files with samtools(*61*) (v 1.4; RRID:SCR_002105), and indexed with samtools(*61*) (v 1.9; RRID:SCR_002105). Normalized bigwig coverage tracks were made with bamCoverage (deepTools (*79*) v 2.5.4; RRID:SCR_016366), with RPKM normalization. For more details, please refer to the pipeline Snakefile_Neun_cutnrun in the github https://github.com/raquelgarza/GBA_snRNAseq_cutnrun_Gustavsson2022.git.

TRANSLATION OF NOVEL TRANSCRIPTS

Structure predictions

Protein sequences of the different isoforms were aligned pairwise to MANE select with BioPython using a BLOSUM62 scoring matrix with gap open penalty of -3 and gap extend penalty of -0.1. pLDDT scores for residues from AlphaFold2 models were extracted and mapped onto the sequence of MANE select according to the alignment. While the structure of the predictions of newly detected isoforms follows mostly the known GBA1 structure a noteworthy breakdown of the confidence score in regions with deletions is visible. This might indicate a conflict between coevolution information and structural templates from dominant isoforms vs. the learned physico-chemical properties of protein structures, which might be *unfavorable* in those regions.

Cell culture

H4 cells (ATCC® HTB-148148™) with homozygous knockout of GBA1 (ENSG00000177628) were generated using indels-based CRISPR/Cas9 technology [gRNA 5’-TCCATTGGTCTTGAGCCAAG-3’ (reverse orientation) targeting exon 7] via Horizon Discovery Ltd. Cells were cultured in DMEM supplemented with 10% foetal bovine serum at 37 °C, 5% CO2. Cells were sub-cultured every 3-4 days at a split ratio of 1:6.

Cell transfection

Cells were transfected using Lipofectamine 3000 reagent (Invitrogen L3000008) according to manufacturer’s instructions. *GBA1* or *GBAP1* transcripts subcloned in the pcDNA3.1(+)-C-DYK vector were designed using the GenSmart design tool and acquired from GenScript.

Western blot

Protein was extracted from whole cells using MSD lysis buffer (MSD R60TX-3) containing 1x cOmplete Mini Protease Inhibitor Cocktail (Roche 11836153001) and 1x PhosSTOP Phosphatase Inhibitor Cocktail (Roche 4906845001). Protein concentration was determined by Bicinchoninic acid (BCA) assay according to manufacturer’s instructions (Pierce 23225). 10-20 µg of protein diluted in NuPAGE™ LDS Sample Buffer (Invitrogen NP0007) and 200 mM DTT was loaded on NuPAGE™ 4-12% Bis-Tris mini protein gels. Gels were run in NuPAGE™ MES SDS Running Buffer (Invitrogen NP0002) at 150V and transferred to 0.2 µm nitrocellulose membranes in Tris-glycine transfer buffer containing 20% MeOH at 30V for 1.5 hrs. Subsequently, membranes were blocked in Intercept Blocking Buffer (LI-COR 927-60001), incubated with primary antibodies overnight at 4 °C, then IRdye-conjugated secondary antibodies before imaging on the LI-COR Biosciences- Odyssey CLx imaging system. Primary antibodies used include mouse anti-FLAG (Sigma F3165), rabbit anti-GBA1 (C-terminal; Sigma G4171) and rabbit anti-GAPDH (Abcam ab9485).

GCase activity assay

Cells cultured on a 96-well plate were washed with PBS (no Ca2+, no Mg2+) and harvested in activity assay buffer containing 50 mM citric acid/potassium phosphate pH 5.0-5.4, 0.25% (v/v) Triton X-100, 1% (w/v) sodium taurocholate, and 1 mM EDTA. After a cycle of freeze/thaw and 30 min incubation on ice, samples were centrifuged at 3,500 rpm for 5 min in 4 °C. Supernatant was collected and incubated in 1% BSA and 2 mM 4-methylumbelliferyl-β-D-galactopyranoside

(4-MUG, Sigma M3633) for 90 min at 37 °C. The reaction was stopped by addition of 1 M glycine pH 12.5, and fluorescence (Ex 365 nm; Em 445 nm) was measured using SpectraMax M2 microplate reader (Molecular Devices). Enzyme activity was normalised to untransfected controls.

Immunofluorescence

Cells cultured on a 96-well plate were fixed in 4% PFA for 10 min, methanol for 10 min, and permeabilized in 0.3% Triton X-100 for 10 min at room temperature. Cells were then blocked in BlockAce blocking reagent (BioRad BUF029) for 60 min then incubated with primary antibodies at 4 °C overnight. Following washing with PBS with 0.1% Tween-20, cells were incubated with Alexa Fluor secondary antibodies and Hoechst nucleic acid stain. Imaging was performed on the Thunder imager (Leica) and Opera Phenix High-content Screening System (PerkinElmer). The proportion of FLAG-tag staining (representing overexpressed GBA1) that localised to lysosomes was quantified using Harmony High-Content Imaging and Analysis Software (PerkinElmer). For each condition, >100 cells were assessed across 2 individual wells with 9 fields of images taken per well. Primary antibodies used include mouse anti-FLAG (Sigma F3165), mouse anti-GBA1 (Abcam ab55080) and rabbit anti-Cathepsin D (Abcam ab75852).

Variant interpretation

We retrieved all genetic variants overlapping the *GBA1* locus from ClinVar, using this script https://github.com/egustavsson/long-read_scripts/blob/main/scripts/getClinVarForLoci.sh and subsequently filtered for only pathogenic variants. Since *GBA1* variants associated with risk of PD are not necessarily classified as pathogenic, we also included data from the GBA1-PD browser (https://pdgenetics.shinyapps.io/gba1browser/)(*80*), a manual curation of PD risk variants in *GBA1*.

Mass spectrometric analysis of prefrontal cortex proteomes

Public mass spectrometry dataset was retrieved from ProteomeXchange (PXD026370) and from MassIVE (MSV000085698). PXD026370 consists of human brain tissue was collected post-mortem from patients diagnosed with multiple system atrophy (*n* = 45) and from controls (*n* = 30) to perform a comparative quantitative proteome profiling of tissue from the prefrontal cortex

(Broadman area 9)(*48*). MSV000085698 consists of label-free mass spectrometry analysis of human ESC–derived microglia-like cell lines (hMGLs)(*81*).

The data analysis was performed using MetaMorpheus(*82*) (v 0.0.320; https://github.com/smith-chem-wisc/MetaMorpheus). The search was conducted for two 2 GBAP1 isoforms (PB.845.1693 and PB.845.525), and a list of 267 frequent protein contaminants found within mass spectrometry data as provided by MetaMorpheus. An FDR (false discovery rate) of 1% was applied for presentation of PSMs (peptide spectrum matches), peptides, and proteins following review of decoy target sequences.

The following search settings were used: protease = trypsin; maximum missed cleavages = 2; minimum peptide length = 7; maximum peptide length = unspecified; initiator methionine behavior = Variable; fixed modifications = Carbamidomethyl on C, Carbamidomethyl on U; variable modifications = Oxidation on M; max mods per peptide = 2; max modification isoforms = 1024; precursor mass tolerance = ±5.0000 PPM; product mass tolerance = ±20.0000 PPM; report PSM ambiguity = True.

ANNOTATION OF PARENT GENES AND PROTEIN-CODING GENES

To explore inaccuracies in annotation of parent genes and protein-coding genes we applied three independent approaches:

Long-read RNA-sequencing

To identify full-length transcripts with at least one novel splice junction we used the same long-read RNA-seq samples available from ENCODE(*56*) as previously described. Transcripts with novel splice junction resulting in novel ORF were those transcripts that had a predicted ORF that was not present in GENCODE v38 annotation.

Novel expressed regions

Novel unannotated expression(*37*) was downloaded from Visualisation of Expressed Regions (vizER; https://rytenlab.com/browser/app/vizER). The data originates from RNA-seq data in base-level coverage format for 7,595 samples originating from 41 different GTEx tissues. Cell lines, sex-specific tissues, and tissues with 10 samples or below were removed. Samples with large chromosomal deletions and duplications or large copy number variation previously associated with disease were filtered out (smafrze = "USE ME"). Coverage for all remaining

samples was normalized to a target library size of 40 million 100-bp reads using the area under coverage value provided by recount2(*55*). For each tissue, base-level coverage was averaged across all samples to calculate the mean base-level coverage. GTEx junction reads, defined as reads with a non-contiguous gapped alignment to the genome, were downloaded using the recount2 resource and filtered to include only junction reads detected in at least 5% of samples for a given tissue and those that had available donor and acceptor splice sequences.

Splice junctions

To identify novel junctions with potential evidence of incomplete annotation, we used data provided by IntroVerse(*83*).

IntroVerse is a relational database that comprises exon-exon split-read data on the splicing of human introns (Ensembl v105) across 17,510 human control RNA samples and 54 tissues originally made available by GTEx and processed by the recount3 project(*33*). RNA-seq reads provided by the GTEx v8 project were sequenced using the Illumina TruSeq library construction protocol (non-stranded 76bp-long reads, polyA+ selection). Samples from GTEx v8 were processed by recount3 through Monorail (STAR(*59*)) to detect and summarise splice junctions and Megadepth(*84*) to analyse the bam files produced by STAR). Additional quality-control criteria applied by IntroVerse included: (i) exclusively analysing samples passing the GTEx v8 minimum standards (smafrze != "EXCLUDE"); (ii) discarding any split-reads overlapping any of the sequences included in the ENCODE Blacklist(*85*); (iii) or split reads that presented an implied intron length shorter than 25 base pairs.

Second, we extracted all novel donor and acceptor junctions that had evidence of use in >=5% of the samples of each tissue and grouped them by gene. We then classify those genes either as "parent" or "protein-coding." Finally, we calculated the proportion that each category of genes presented within each tissue. Focusing on the *parent genes* category, this can be described as it follows:

$$P_T^j = \frac{j}{x}$$

Let j denote the total number of parent genes containing at least one novel junction shared by >=5% of the samples of the current tissue. Let x denote the total number of *parent* genes available for study. Let T denote the current tissue.

We mirrored the formula above to calculate the proportion of protein-coding genes per tissue.

FIGURE GENERATION

The code for all figures in this manuscript can be accessed through:
https://github.com/egustavsson/GBA_GBAP1_manuscript.git

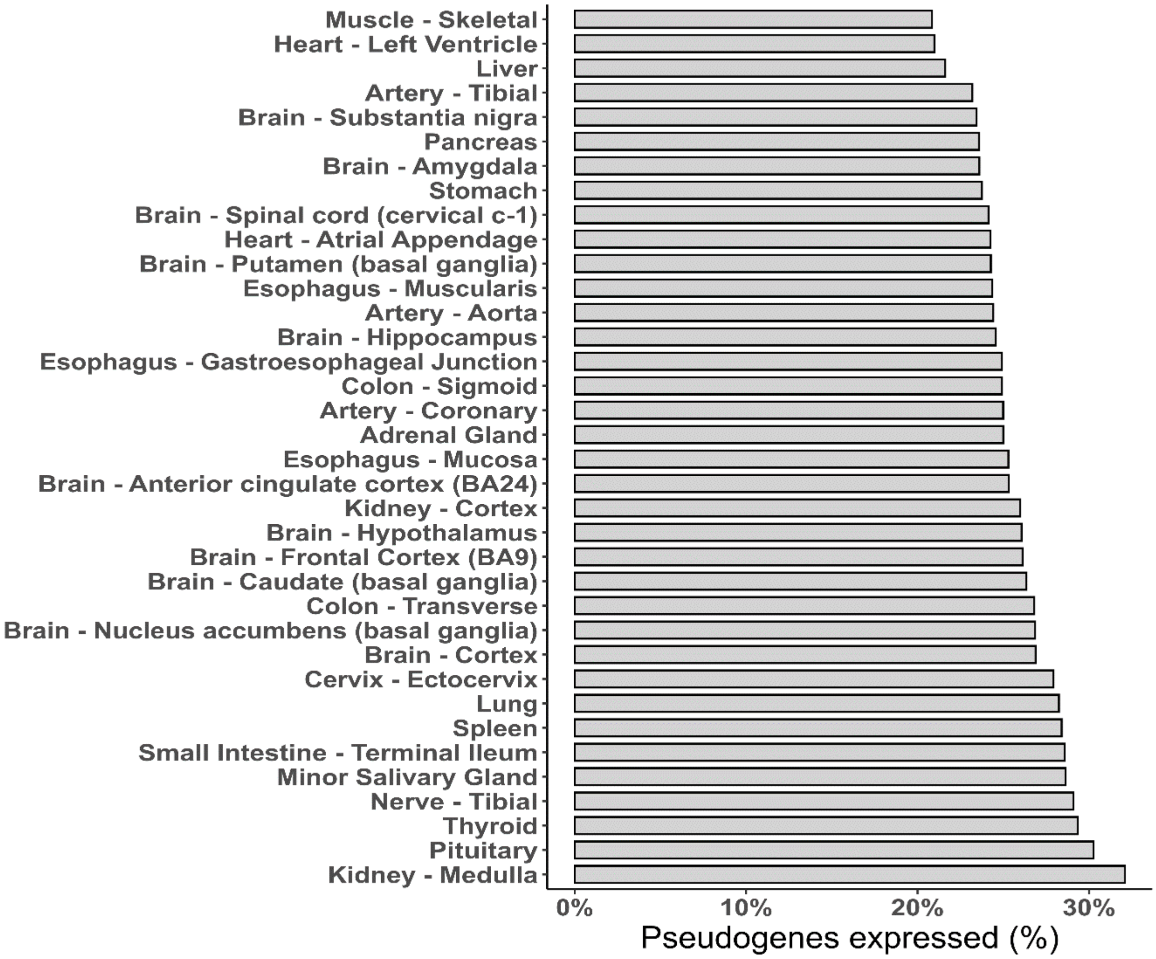

Supplementary Fig. 1: Widespread expression of Pseudogenes across human tissues.
A histogram illustrating the prevalence of expressed pseudogenes in various human tissues, assessed through uniquely mapped reads generated by the Genotype-Tissue Expression Consortium (GTEx v8). The percentage on the X-axis represent the proportion of Pseudogenes that were expressed. A median Transcripts Per Million (TPM) > 0 was required for a Pseudogene to be considered to be expressed.

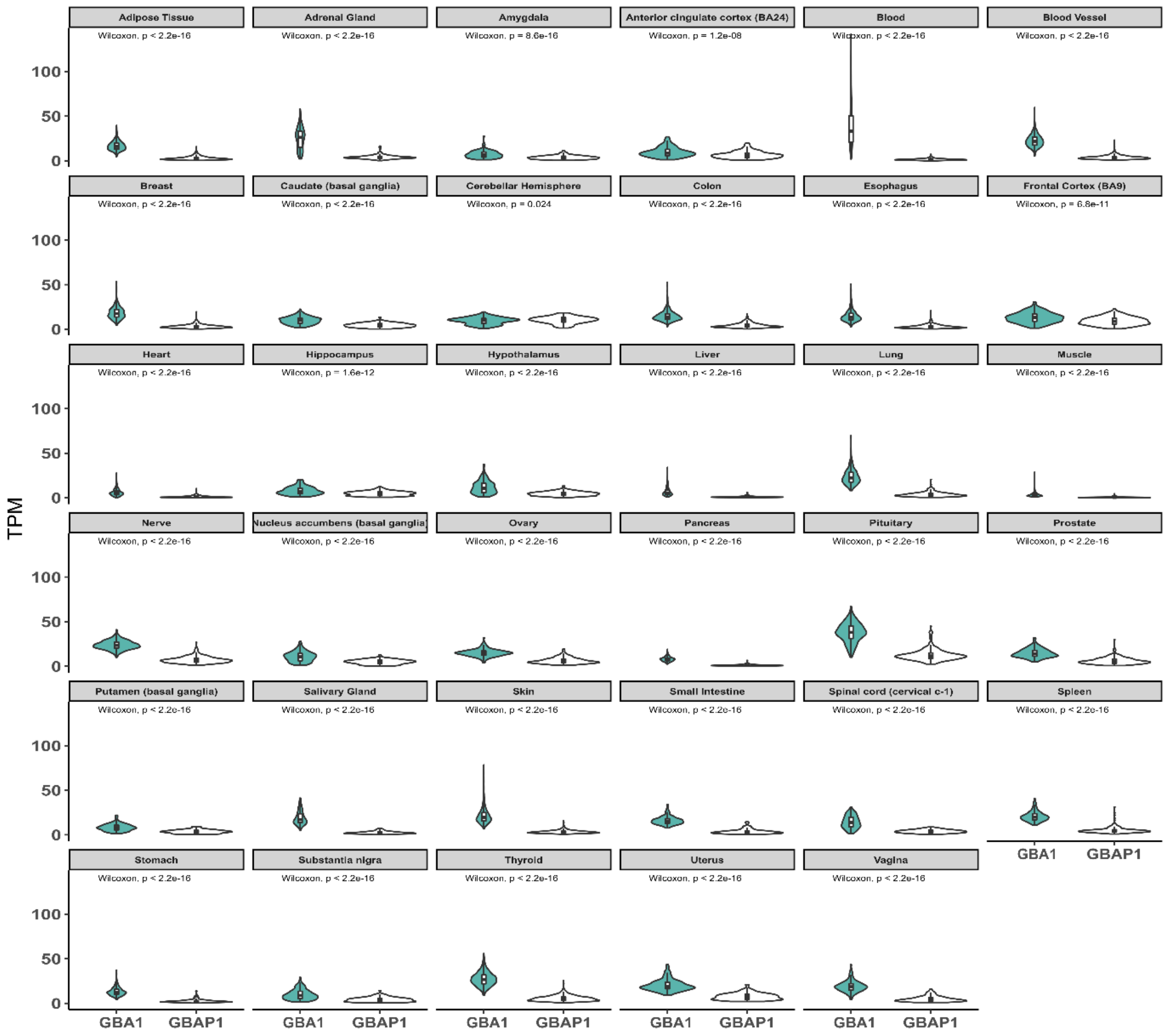

Supplementary Fig. 2: Widespread expression of *GBA1* and *GBAP1* across human tissues. Violin plots depicting the widespread short-read RNA-sequencing expression of *GBA1* (turquoise) and *GBAP1* (white) across 35 human tissues. Expression is measured in transcripts per million (TPM) and data were generated by the Genotype-Tissue Expression Consortium, GTEx v8).

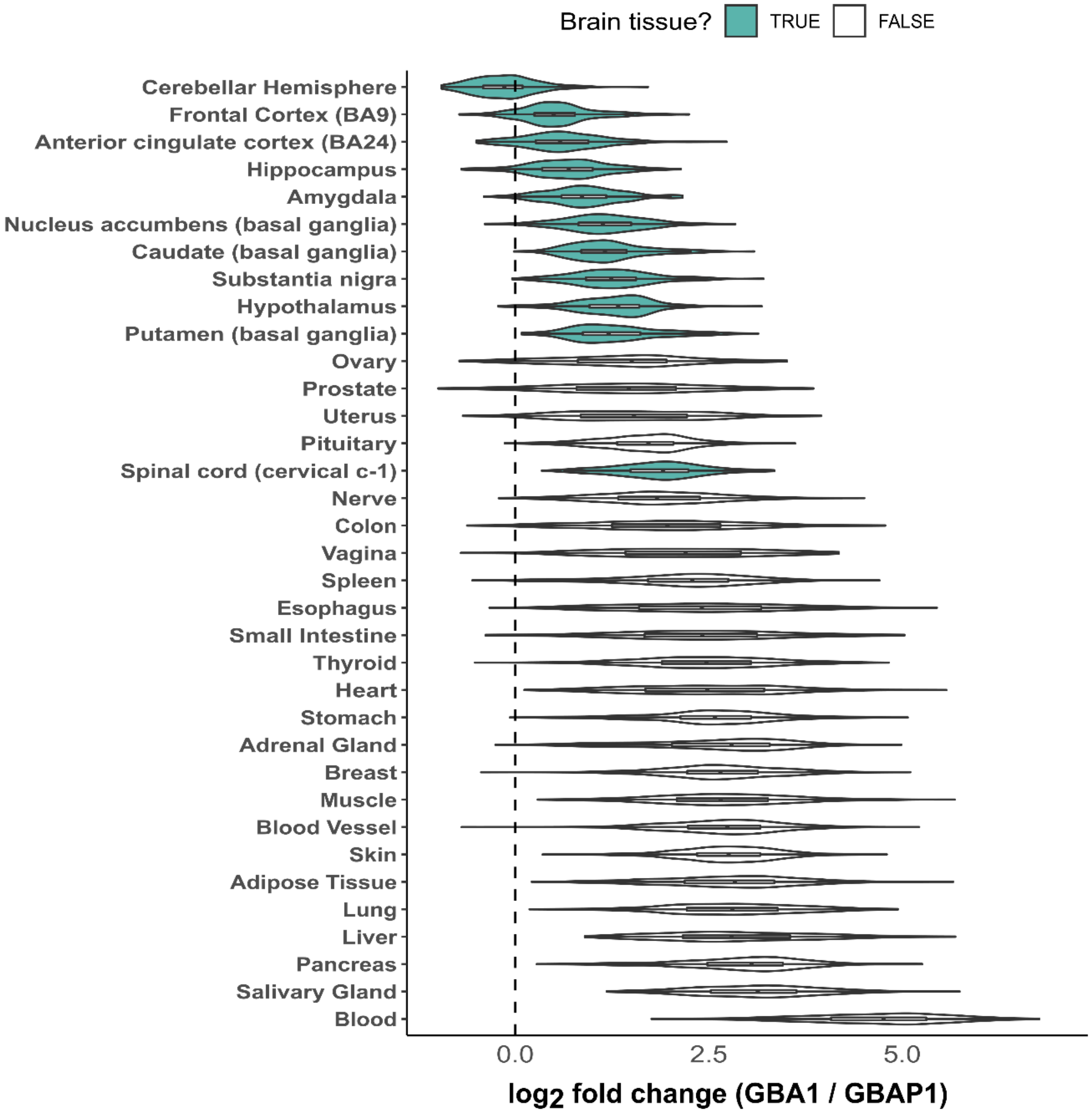

Supplementary Fig. 3: Comparative analysis of *GBA1* and *GBAP1* expression across human tissues.

Violin plots illustrating the $\log_2$ fold change of *GBA1* (numerator) relative to *GBAP1* (denominator) across human tissues. Values above 0 indicate higher *GBA1* expression relative to *GBAP1* whereas values below 0 indicates higher *GBAP1* expression relative to *GBA1*. Brain tissues are filled in turquoise. Expression data (transcripts per million [TPM]) derived from the Genotype-Tissue Expression Consortium, GTEx v8).

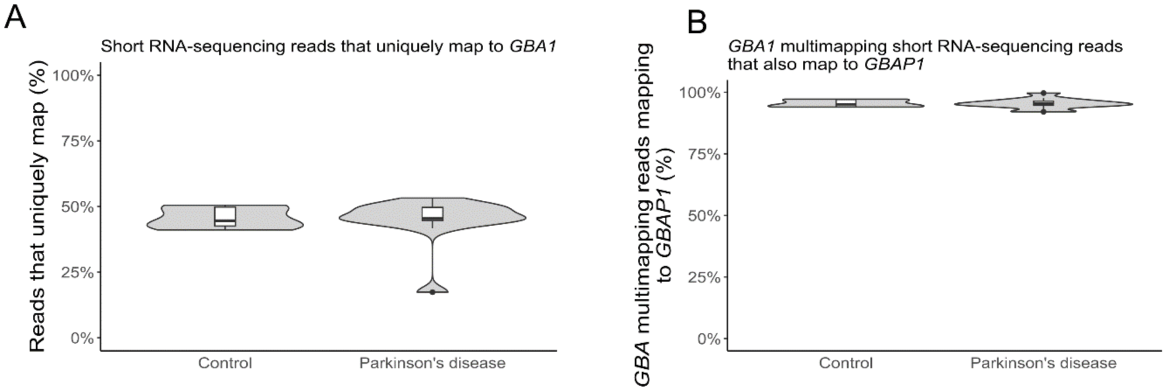

Supplementary Fig. 4: Multimapping analysis of short RNA-sequencing reads that map to *GBA1*. (**A**) Violin plots showing multimapping of *GBA1* from short-read RNA-seq data (100 bp paired end reads, mean reads per sample of 182.9 ± 14.9M) from human post-mortem anterior cingulate cortex samples generated from control (n = 5) and PD-affected individuals (n = 7)**(*8*)**. (**B**) Violin plots showing the percentage of *GBA1* short RNA-sequencing multimapping reads that that also map to *GBAP1*.

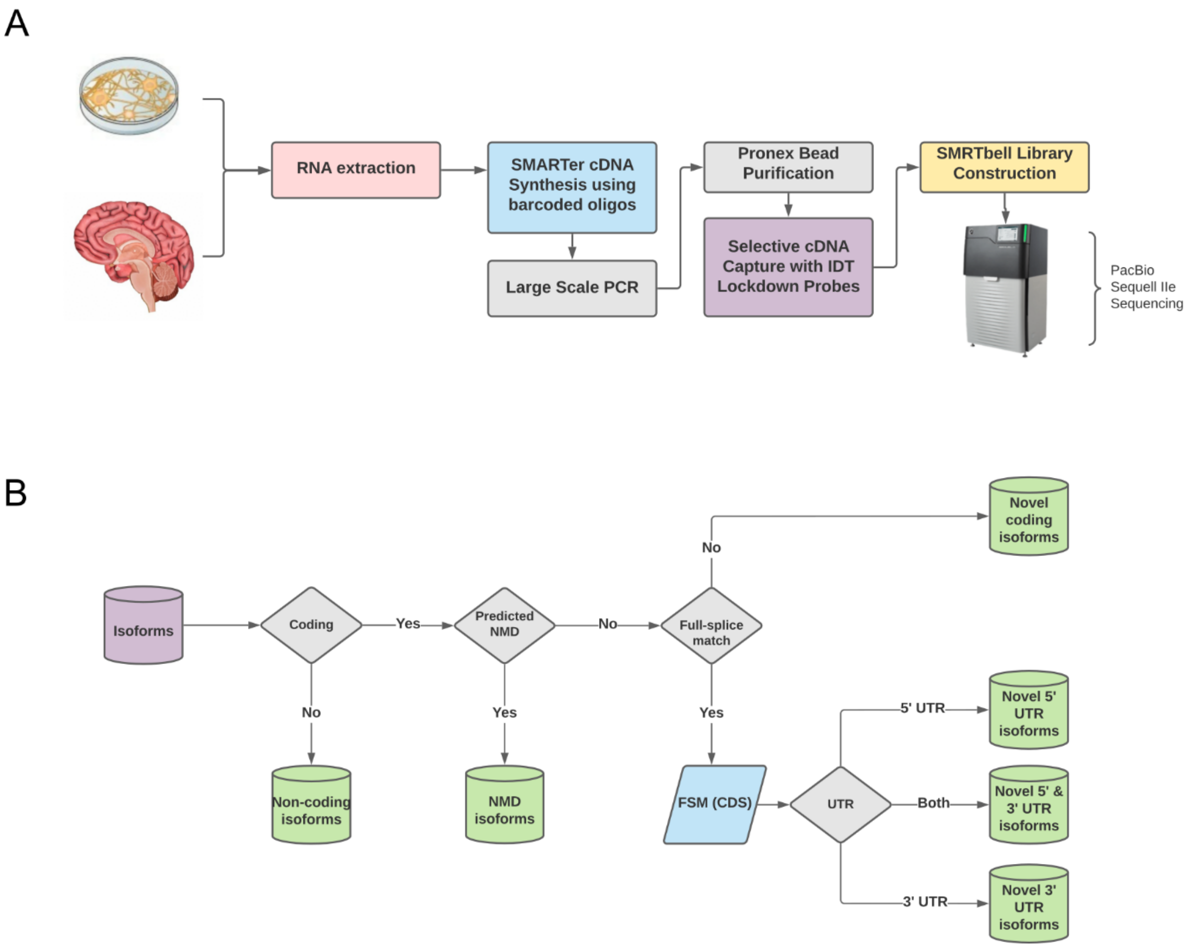

Supplementary Fig. 5: Targeted Long-Read RNA Sequencing Strategy.

(**A**) Schematic representation outlining the methodology employed for targeted long-read RNA sequencing of *GBA1* and *GBAP1* across diverse human brain tissues and cell types, including neurons derived from induced pluripotent stem cells (iPSC), microglia, and astrocytes.

(**B**) A flowchart delineating the categorization process of transcripts obtained through long-read RNA sequencing. This approach provides a systematic overview of the strategy used to capture and analyze the expression profiles of *GBA1* and *GBAP1* in various cell populations within the human brain.

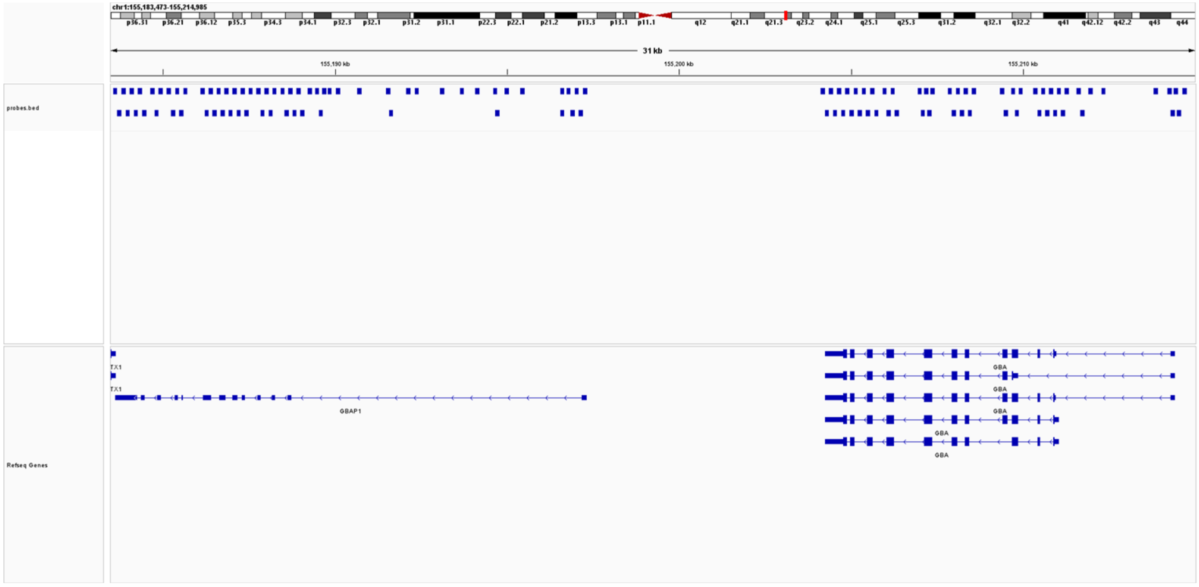

Supplementary Fig. 6: ***GBA1*** **and** ***GBAP1*** **hybridization probe design.**

Enrichment of *GBA1* and *GBAP1* was done using 119 biotinylated IDT lockdown hybridization probes. This The Integrated Genomics Viewer (IGV) window shows the location of the 120-mer hybridization probe design used for the enrichment of *GBA1* (n = 54) and *GBAP1* (n = 65) cDNA.

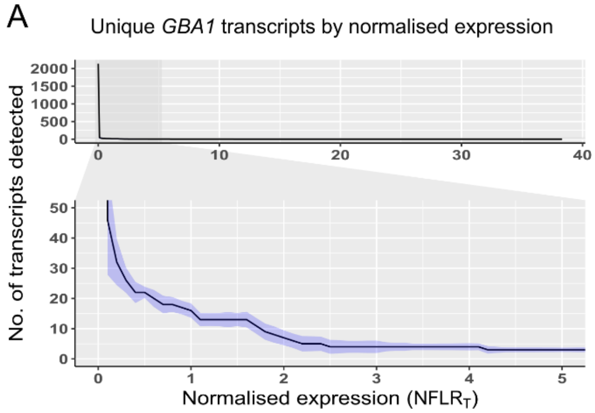

Supplementary Fig. 7: Total number of unique transcripts of *GBA1* and *GBAP1* by normalized expression. (**A**) Depreciation curve showing the number of unique *GBA1* transcripts on the Y-axis increased by increasing the normalized full-length read count of transcript ($NFLR_T$) on the X-axis. $NFLR_T$ is the total number of reads per transcript normalized by the total number of reads of the loci. (**B**) Depreciation curve showing the number of unique *GBAP1* transcripts on the Y-axis increased by increasing the $NFLR_T$ on the X-axis.

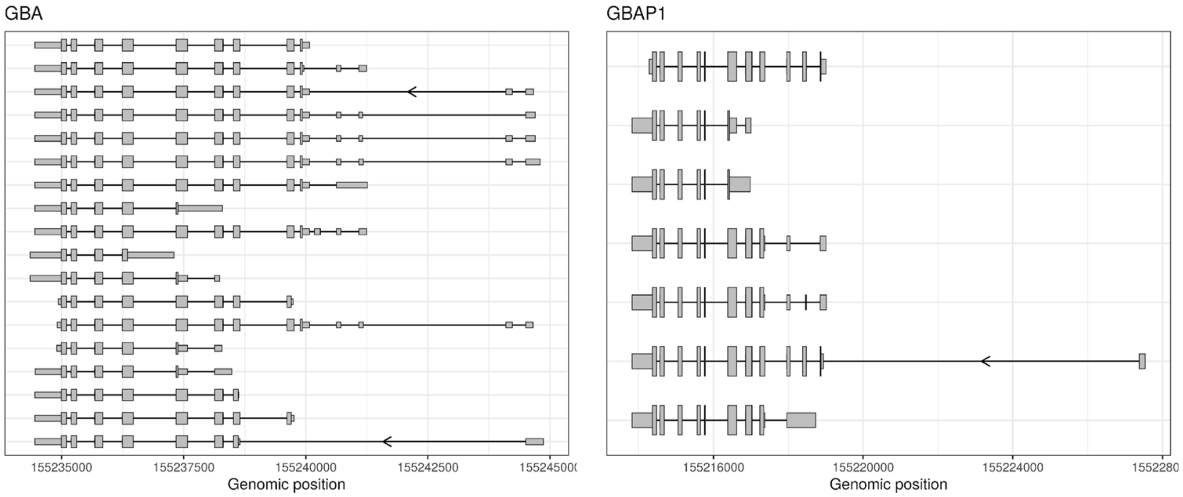

Supplementary Fig. 8: *GBA1* and *GBAP1* transcripts with novel open reading frames. A total of 18 *GBA1* transcripts with novel open reading frames (ORF) identified through targeted long-read RNA sequencing of 12 human brain regions on the left. A total of 7 *GBAP1* transcripts with ORFs predicted to be coding identified through targeted long-read RNA sequencing of 12 human brain regions on the right.

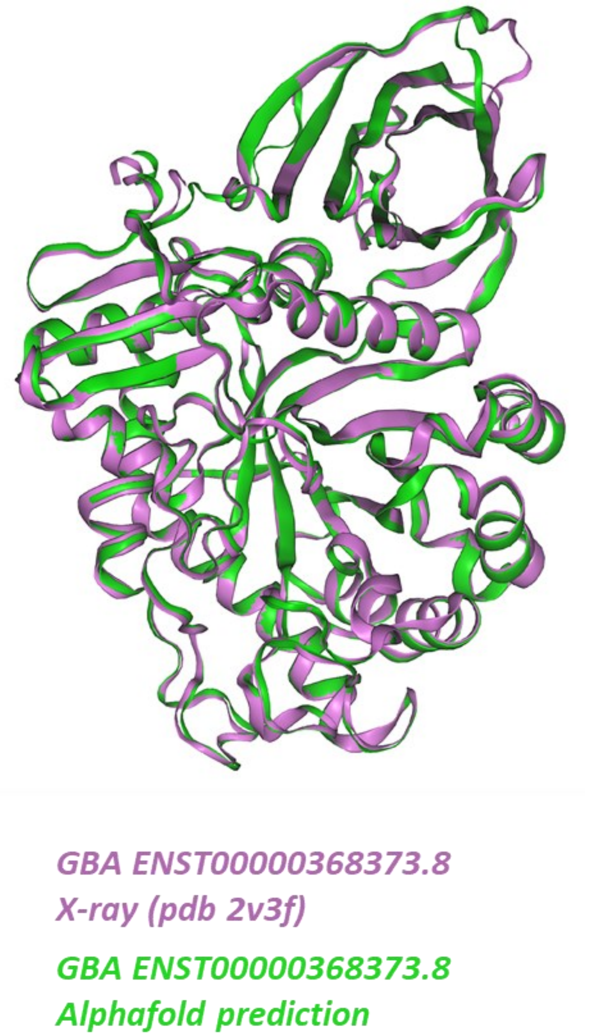

Supplementary Fig. 9: Structure of GBA1. Experimental X-ray structure of MANE select (PDB ID 2v3f) (violet) superimposed on AlphaFold2 prediction of the same sequence (green). This illustrates the accuracy of which AlphaFold2 predicts the structure of GBA1.

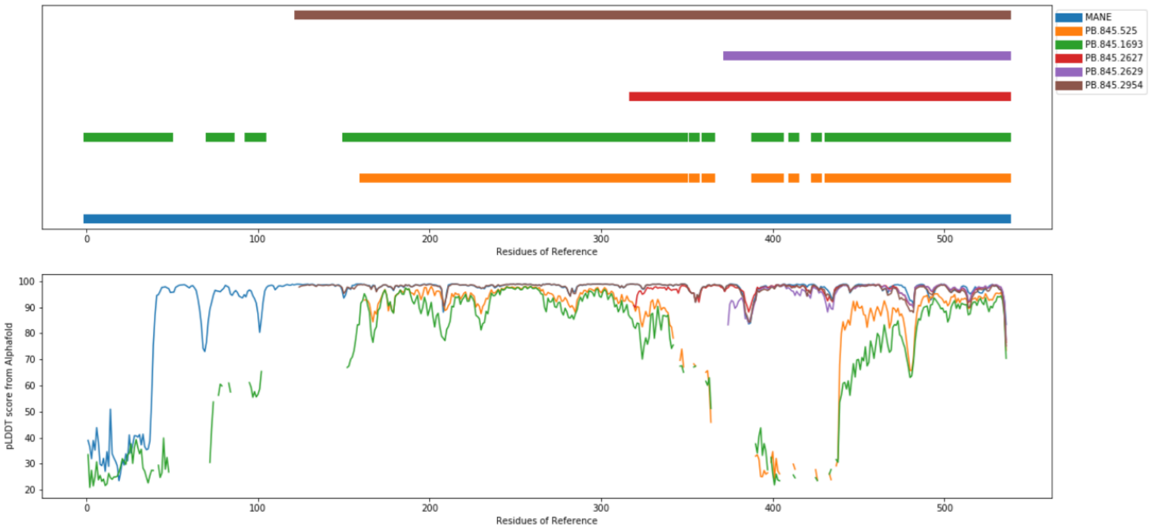

Supplementary Fig. 10: Pairwise alignment of novel GBA1 and GBAP1 peptide sequences. Pairwise alignment of novel peptide sequences from GBA1 isoforms (PB.845.2954 in brown, PB.845.2627 in red and PB.845.2629 in purple) and GBAP1 (PB.845.525 in orange and PB.845.1693 in green) against the GBA1 MANE select isoform (ENST00000368373 in blue). The top panel illustrates the alignment where a gap in one of the sequences means that one or more amino acid residues are missing from the sequence as compared to GBA1 MANE select isoform (ENST00000368373 in blue) and the bottom panel shows the per-residue model confidence score (pLDDT) which ranges between 0 and 100.

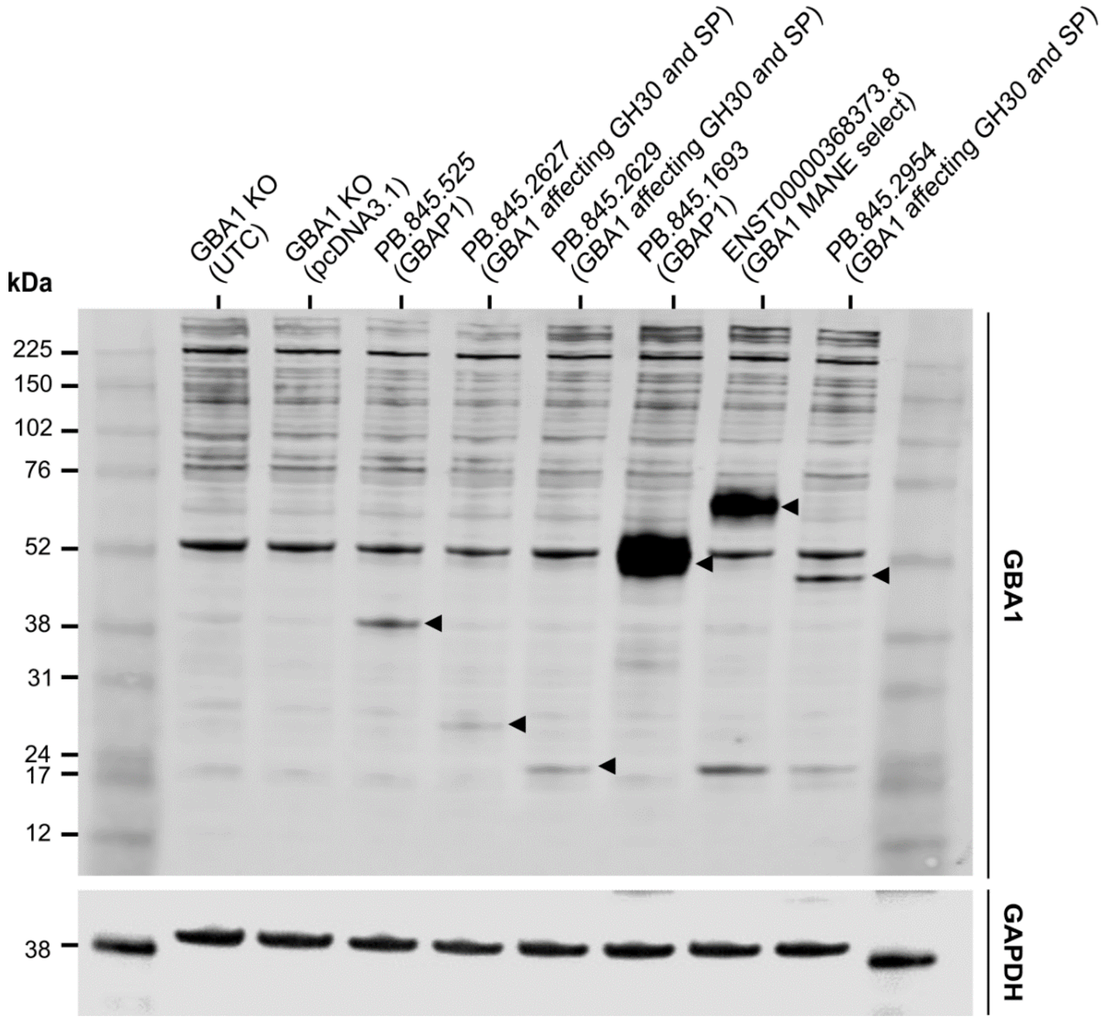

Supplementary Fig. 11: Detection of novel protein GBA1 and GBAP1 isoforms with a anti-GBA1 C-terminus antibody. Immunoblot of H4 GBA1(-/-/-) knockout cells transiently transfected with *GBA1* and *GBAP1* constructs containing a c-terminus FLAG-tag. GBA1 and GBAP1 expression was detected using GBA1 anti-rabbit G4171 from Sigma-Aldrich (1:1000), synthetic peptide corresponding to amino acids 517-536 (c-terminus) of human glucocerebrosidase conjugated to KLH. GAPDH was used as a loading control. The predicted protein sizes are: PB.845.525 (GBAP1; 321 aa; 35 kDa), PB.845.2627 (GBA1 affecting GH30 and SP; 219 aa; 24 kDa), PB.845.2629 (GBA1 affecting GH30 and SP; 164 aa; 18 kDa), PB.845.1693 (GBAP1; 399 aa; 44 kDa), ENST00000368373 (GBA1 MANE select; 537 aa; 62 kDa) and PB.845.2954 (GBA1 affecting GH30 and SP; 414 aa; 46 kDa).

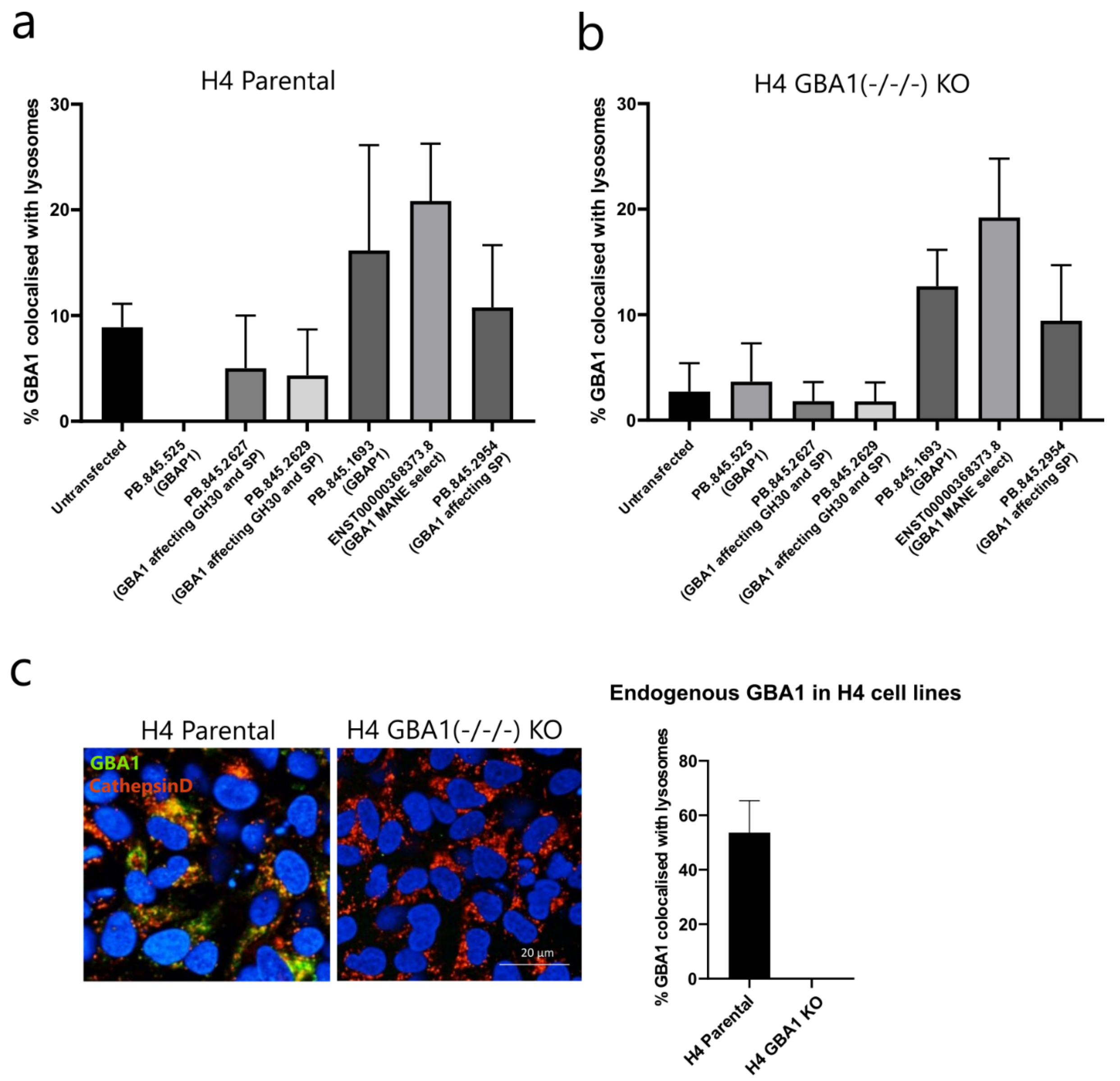

Supplementary Fig. 12: Lysosomal colocalization of *GBA1* and *GBAP1* transcripts. Immunohistochemistry of H4 parental (**A**) and GBA1(-/-/-) knockout cells (**B**) transiently transfected with GBA1 and GBAP1 constructs containing a c-terminus Flag tag. Proportion of GBA1-FLAG or GBAP1-FLAG (Green) that colocalised with CathepsinD (Red) was quantified as a percentage of all GBA1-FLAG or GBAP1-FLAG staining. (**C**) Immunohistochemistry of H4 parental and GBA1(-/-/-) knockout cells. Proportion of anti-GBA1 (Green) that colocalised with anti-Cathepsin D (Red) was quantified as a percentage of all anti-GBA1 staining using mouse anti-GBA1 (Abcam ab55080) and rabbit anti-Cathepsin D (Abcam ab75852).

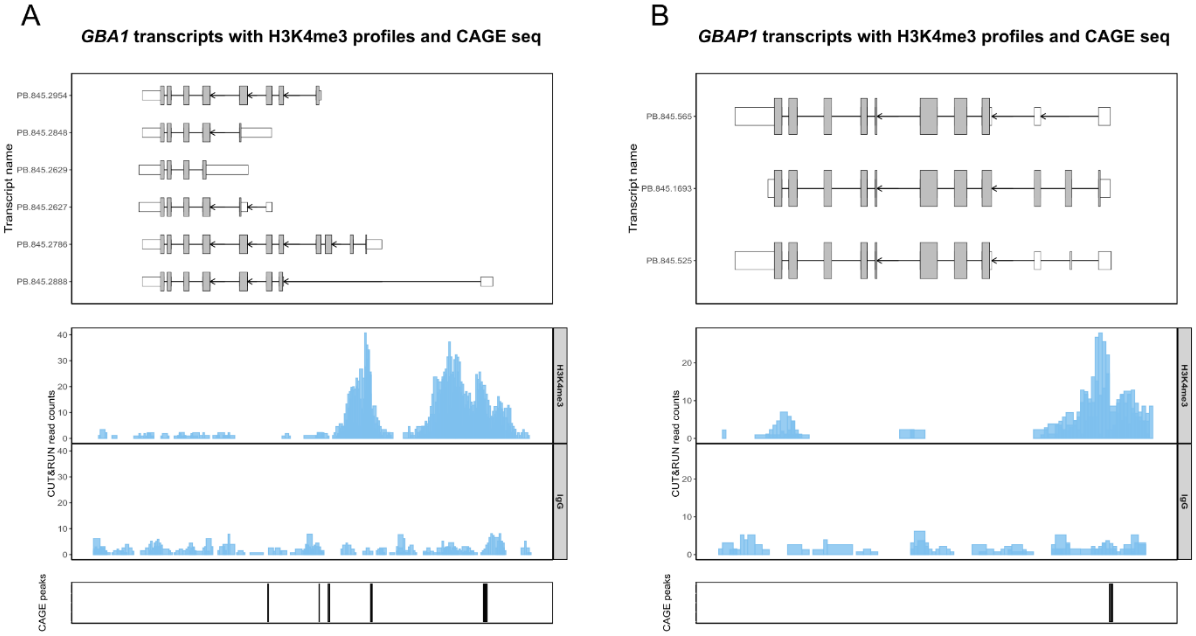

Supplementary Fig. 13: Transcriptionally active euchromatin at the 5' TSS of GBAP1 ORF transcripts. (**A**) Novel protein coding transcripts of GBA1 CUT&RUN profiling of H3K4me3 marks in neurons (based on NeuN+) and CAGE sequencing data from FANTOM5. (**B**) Novel protein coding transcripts of GBAP1 CUT&RUN profiling of H3K4me3 marks in neurons (based on NeuN+) and CAGE sequencing data from FANTOM5.

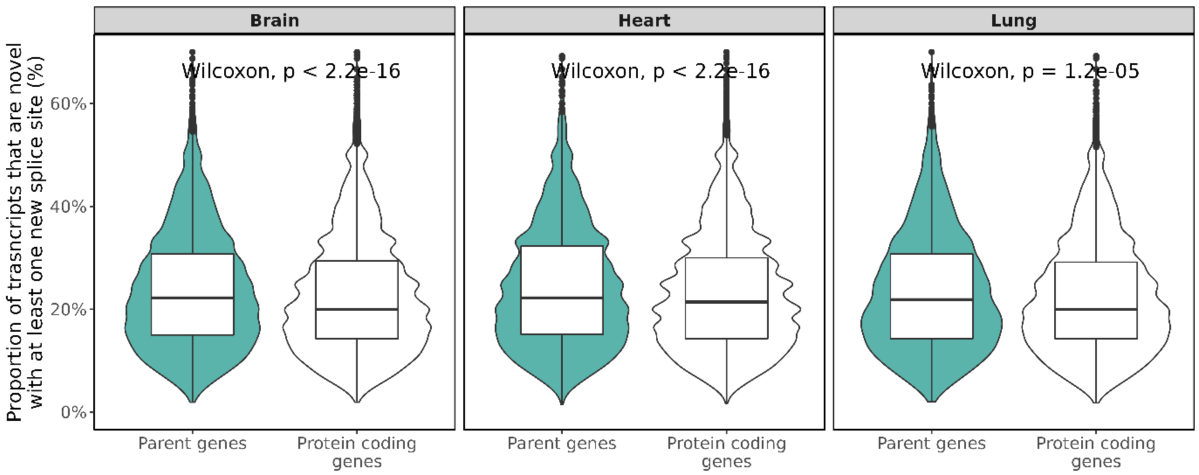

Supplementary Fig. 14: Inaccuracies in annotation is common for parent genes on a genome-wide scale across tissues. Proportion of transcripts per parent gene and per protein coding gene without a pseudogene with a novel splice site from long-read RNA-sequencing data in Brain (n = 9), Heart (n = 16) and lung (n = 6).

Table S1.
Parent genes and pseudogenes

Table S2.
Brain regions sequenced

Table S3.
Public long-read RNA sequencing data included

Table S4.
Barcodes used for multiplexing samples during targeted long-read RNA sequencing

Data S1. (separate file)
Supplementary tables S1-S4 as an XLSX file.
